# Genetic–epigenetic interactions in *cis*: a major focus in the post-GWAS era

**DOI:** 10.1186/s13059-017-1250-y

**Published:** 2017-06-19

**Authors:** Catherine Do, Alyssa Shearer, Masako Suzuki, Mary Beth Terry, Joel Gelernter, John M. Greally, Benjamin Tycko

**Affiliations:** 1 Institute for Cancer Genetics and Herbert Irving Comprehensive Cancer Center, Columbia University, New York, NY 10032 USA; 2 Center for Epigenomics, Department of Genetics, Albert Einstein College of Medicine, Bronx, NY 10461 USA; 3 Department of Epidemiology, Columbia University Mailman School of Public Health, and Herbert Irving Comprehensive Cancer Center, Columbia University, New York, NY 10032 USA; 4 Departments of Psychiatry, Genetics, and Neurobiology, Yale University School of Medicine, New Haven, CT 06520 USA; 5 Institute for Cancer Genetics, Herbert Irving Comprehensive Cancer Center, Taub Institute for Research on Alzheimer’s disease and the Aging Brain, New York, NY 10032 USA; 6 Department of Pathology and Cell Biology, Columbia University, New York, NY 10032 USA

## Abstract

Studies on genetic–epigenetic interactions, including the mapping of methylation quantitative trait loci (mQTLs) and haplotype-dependent allele-specific DNA methylation (hap-ASM), have become a major focus in the post-genome-wide-association-study (GWAS) era. Such maps can nominate regulatory sequence variants that underlie GWAS signals for common diseases, ranging from neuropsychiatric disorders to cancers. Conversely, mQTLs need to be filtered out when searching for non-genetic effects in epigenome-wide association studies (EWAS). Sequence variants in CCCTC-binding factor (CTCF) and transcription factor binding sites have been mechanistically linked to mQTLs and hap-ASM. Identifying these sites can point to disease-associated transcriptional pathways, with implications for targeted treatment and prevention.

## Introduction

The ongoing debate on “nature versus nurture” in determining human traits and diseases provides a useful framework for making sense of a growing mass of genomic and epigenomic data. Although environmental influences such as nutrition, stress, and chemical exposures (“nurture”) can alter epigenetic marks, we focus here on genetic influences (“nature”) in determining epigenetic patterns. With the discovery and mapping of haplotype-dependent allele-specific DNA methylation (hap-ASM; Table 1) and methylation quantitative trait loci (mQTLs; also known as meQTLs; Table 2), studies on *cis*-acting genetic–epigenetic interactions are proliferating. Furthermore, such studies are becoming highly relevant as we move into the post-genome sequencing and post-genome-wide-association-study (post-GWAS) era. Mapping of ASM and mQTLs is being developed as a method for pinpointing DNA sequence variants that underlie genetic susceptibility to common diseases, ranging from cardiovascular and metabolic disorders to neurodegenerative and neuropsychiatric diseases, autoimmune conditions, and cancers. Such mapping is helping to overcome major roadblocks in GWAS that arise from the fact that most GWAS peaks map to non-protein-coding sequences, where their molecular consequences can be difficult to evaluate. Conversely, ASM and mQTLs must be identified and filtered out when searching for (non-genetic) effects of environment and disease progression in epigenome-wide association studies (EWAS).

**Table 1:** Methods and conclusions from studies of hap-ASM

Tissues or cell types (*n*)	Hap-ASM: primary screening method and validations	Findings and conclusions	Reference
PBL (6), placenta (3), other normal tissues (7)	MSNP Affy 50 K/250 K; validation by pre-digestion/PCR assays and bis-seq	58 candidate ASM loci identified; 12/16 selected loci independently validated. For a given locus, hap-ASM was seen in 95 to 40% of heterozygotes. ASM in *CYP2A7* and *VNN1* associated with ASE	[48]
PBL (38)	Targeted bis-seq; validation by *Hpa*II pre-digestion/Seq	ASM found in ~10% of CGIs on Hsa21. For a given locus, ASM was seen in 95 to 13% of heterozygotes; ASM associated with ASE in *C21orf81*	[182]
LCL (13), PBL (3)	MSNP Affy 500 K; validation by bis-seq	~10% of queried CpGs showed a *cis*-effect. In some cases, there was a short-range effect of CpG SNPs on methylation at nearby non-polymorphic CpGs	[183]
hESC (3), fibro (4), fibro-reprogrammed iPS cells (5), fibro-derived lymphocytes (3), hESC-fibro hybrid cell (1)	Bis-seq with padlock probes; validation by targeted bis-seq	Non CpG-SNP ASM DMRs were observed in 3–22% of the queried regions; half of these DMRs contained both CpG-SNPs and bona fide ASM. ASM validated in 5/12 selected loci	[59]
PBL (10), buccal cells (10)	MSNP Affy 6.0 array; validation by bis-seq and MS-SNuPE. eQTLs assessed using Affy U133 chips	~1.5% of CpGs showed ASM; 16.3% of the ASM were within 5 kb of a gene that was associated with an eQTL	[184]
PBMC of one individual	WGBS, ASE by TA clone sequencing	599 ASM DMRs with an average size of 312 bp were identified; 5/6 selected genes with haploid DMR(s) within 2 kb of their TSS were associated with ASE	[60]
PBL (8), LCL (1), hESC (1), kidney (1), muscle (1)	RRBS; PCR-based bis-seq validation; RNA-Seq for ASE	~8% of SNPs associated with ASM. ASM regions depleted in CGIs, located in intergenic regions with low evolutionary conservation; enriched in genes with ASE	[61]
PBL (42)	MSNP Affy 6.0 array; validation by bis-seq	Hap-ASM in ~5% of the CpGs; inter-individual variation; multiple hap-ASM SNPs found in LD with GWAS peaks for immune/inflammatory diseases	[63]
Liver (20), brain (13), placenta (20), PMN (5), PBL (22), PBMC (15), lung (7), heart (4), breast epithelial cells (5), sperm (2)	MSNP Affy 250 K and 6.0 arrays; bis-seq for validation and fine-mapping	Mapping of hap-ASM DMRs in *STEAP3* and *CYP2A7* and imprinted ASM in *VTRNA2* and *RPN1* showed discrete DMRs precisely overlapping CTCF-binding sites. *STEAP3*, *CYP2A7* and *RPN1* show ASE	[51]
PBL (96) from parent–child trios	Bis-seq with padlock probes; Illumina 550 K arrays; Affy 6.0 arrays	Mid-parent offspring, mQTL and ASM analyses revealed *cis*-acting effects on ~5–14% of the queried CpGs; inter-individual variation in hap-ASM	[185]
Brain (3), T cells (3), liver (2), placenta (2), fetal heart (2), fetal lung (1), macaque PBL and liver (4)	Agilent Methyl-seq, validation by targeted bis-seq and ox-bis-seq	Hap-ASM in ~2% of informative regions; 188 DMRs located near GWAS signals for immune or neuropsychiatric disorders. Hap-ASM DMRs enriched in polymorphic CTCF sites and TFBS. CTCF- and TF-binding likelihood predicts strength and direction of hap-ASM	[49]
145 CD4+ T cells (145), VAT (148), WB (599), monocytes (12), muscle (6)	MCC-seq; WGBS for ASM and mQTL; validation by Illumina 450 K Methyl, genotyping by WGS, Illumina Omni2.5 M, Omni5M; RNA-seq for ASE; ChIP-seq for ASH	Of ~2.2 M queried CpGs, ~32% showed ASM or mQTLs, and ~14% of CpGs showing methylation asymmetry without a genetic basis. 25% and >50% of the instances of ASM and mQTLs, respectively, were tissue-specific. ASM and mQTLs were enriched in enhancers; SNPs linked to ASH were enriched for association with ASM	[53]

**Table 2:** Methods and conclusions from studies of cis-acting mQTLs

Tissues or cell types (*n*)	mQTLs: primary screening method and validations	Findings and conclusions	Reference
Cerebellum (153)	Illumina 27 K Methyl; Affy 5.0 SNP chips; validation by Pyroseq; eQTLs:Affy HGU95Av2	mQTLs detected at ~8% of the CpGs; mQTL CpGs enriched in CGIs and within 150 kb of the index SNP; 13% of mQTL index SNPs associated with eQTLs	[62]
Brains (150 individuals; 4 brain regions)	Illumina 27 K Methyl; Illumina 550 K SNP chips; eQTLs: Illumina HumanRef-8	mQTLs detected at ~5% of CpGs. mQTL CpGs were depleted in CGIs. ~50% of the mQTLs were detected only in one brain region. ~5% of the index SNPs were both mQTLs and eQTLs	[108]
Adipose tissue (648), replication set PBL (200)	Illumina 450 K Methyl; multiple genotyping arrays, eQTLs: HT-12 V3 BeadChips; validations by WGBS	mQTLs detected at ~28% the CpGs, with tissue-specificity; 22% of eQTLs were in LD with at least one mQTL; ~4% were in LD with a GWAS SNP; mQTLs associated with eQTLs and GWAS SNPs were enriched in enhancers	[110]
Cord blood (174), PBL (90), TC (125), FC (111), pons (106), cerebellum (105)	Illumina: 27 K Methyl BeadChips; multiple Illumina and Affy genotyping arrays	mQTLs detected at ~5% of the CpGs; overlap observed between ancestral groups, developmental stages, and tissue types; brain mQTL SNPs enriched in bipolar disorder GWAS peaks and miRNA-binding sites	[155]
TC (44), neurons (18), glia (22), T-cells (54), placenta (37)	Illumina 450 K Methyl and 2.5 M SNP chips; validation by bis-seq and ox-bis-seq	~3000 strong mQTLs identified; more than half tissue-restricted and ~900 located near GWAS signals; mQTLs enriched in polymorphic CTCF-binding sites and TFBS, and enriched in eQTLs located within 20 kb	[49]
Fetal brain (166), matched adult PFC, striatum and cerebellum (83)	Illumina 450 K Methyl; 2.5 M SNP chips	Most fetal mQTLs also present in adult brain, but ~1/3 showed differential effects; mQTLs enriched in repressive and poised histone marks; mQTLs enriched in CTCF motifs, eQTLs, and schizophrenia-associated GWAS peaks	[112]
PBL (85)	Illumina 27 K Methyl; OmniExpress SNP chips	1287 smoking associated DM CpGs and 770 mQTLs identified. Among these, 43 CpGs were both smoking DM and mQTL	[150]
Adipose tissue (119)	Illumina 450 K Methyl; Omni SNP chips; eQTL:Affymetrix Human Gene 1.0 ST array	mQTLs detected in ~3% of the CpGs; enriched in CGI shelves and shores and depleted in promoter regions and CGI; ~1% of mQTL SNPs (or proxy) were obesity-associated GWAS SNPs; 2% of the SNPs showed both mQTL and eQTL	[113]
CD4+ T cells (717)	Illumina 450 K Methyl; Affy 6.0 SNP chips	Of ~20,000 heritable CpGs identified by modeling family structure, 15,133 were *cis*-mQTLs; 1329 trans-mQTLs and 4113 CpGs showing no evidence of *cis* or *trans* mQTL	[54]
Monocytes (197), neutrophils (197), and CD4+ T cells (132)	Illumina 450 K Methyl; WGS; RNA-seq for ASE and ChIP-seq for hQTLs	mQTLs affect 10% of CpGs, hQTLs found in 28 and 12% of H3K4me1 and H3K27ac peaks; 345 GWAS index SNPs (or SNPs in high LD with a GWAS index SNPs) colocalized with mQTLs and/or hQTLs	[37]

This list of studies is representative of the historical progression of the field and is not meant to be comprehensive. All experiments include internal statistical validations of the microarray and sequencing data; secondary validations refer to downstream assays by independent methods. Cells and tissues are of human origin unless otherwise stated. *Abbreviations*: *ASH* allele-specific histone modifications, *CGI* CpG island, *FC* frontal cortex, *fibro* fibroblast cell lines, *hESC* human embryonic stem cell, *hQTL* histone modification QTL, *iPS* induced pluripotent stem, *LCL* lymphoblastoid cell line, *MCC-seq* MethylC-Capture sequencing, *MSNP* methylation-sensitive SNP array, *PBL* peripheral blood leukocyte, *PBMC* peripheral blood mononuclear cell, *PFC* prefrontal cortex, *PMN* polymorphonuclear leukocyte, *RRBS* reduced representation bis-seq, *TC* temporal cortex, *TSS* transcription start site, *VAT* visceral adiposis tissue, *WB* white blood cell, *WGS* whole genome sequencing

Here, we review recent work on *cis*-acting genetic–epigenetic interactions, including the genome-wide mapping of ASM, mQTLs, and related types of allele-specific epigenetic marks, such as allele-specific chromatin accessibility and allele-specific transcription factor binding. We also briefly cover the discovery and mapping of expression quantitative trait loci (eQTLs) and allele-specific RNA expression (ASE), and we explain the usefulness of each of these types of allele-specific maps for extracting maximum biological information from GWAS data. We point out useful public databases, and we discuss bioinformatic approaches, cross-species comparisons, and functional assays for investigating the molecular mechanisms that produce allele-specific epigenetic marks. Emerging from these studies is a central role for transcription factor binding site (TFBS) occupancies in shaping allele-specific epigenetic patterns. We argue that a continued focus on defining functional genetic variants in such sites will be crucial for connecting allele-specific epigenomic data to disease pathogenesis.

## Successes from GWAS and challenges for post-GWAS

### GWAS and the “missing heritability” problem

In 2012, Visscher et al. [1] summarized the history of GWAS, focusing on the discoveries made and what those discoveries do and do not reveal about the biology of complex traits and disease susceptibility. From articles by prominent scientists, they identified negative opinions such as “GWAS have been disappointing in not explaining more genetic variation in the population”, and “GWAS have not delivered meaningful, biologically relevant knowledge or results of clinical or any other utility”. In fact, after two decades of work, with substantial funding, GWAS have uncovered numerous reproducible associations of common genetic variants, mostly single nucleotide polymorphisms (SNPs; sometimes called “simple nucleotide polymorphisms” to include small insertion or deletion variants), with human traits and diseases. It is true that the cumulative effects of disease-associated SNPs have failed to account for the majority of complex-trait heritability [2], but mature GWAS data for many diseases now typically account for more than 10% of such heritability, and this information is starting to have clinical applications, particularly when combined into polygenic risk scores. For example, while the odds ratio (OR) for a given SNP genotype at a GWAS peak (the “GWAS index SNP”) is often <1.2 and seldom >1.4, meta-analyses of, for example, cancer GWAS have shown that the combined effects of a large number of susceptibility loci may become large enough to be useful for risk prediction and targeted prevention, including the provision of more frequent screening [3–5]. Similarly, findings from GWAS have helped to advance the field of pharmacogenomics, with implications for individualized therapies [6, 7].

Nonetheless, the “missing heritability” problem raises the question of whether there are additional common DNA variants with smaller effects that are not being identified because they are yielding sub-threshold signals, or whether there are many rare variants with stronger effects, which would not be readily detectable in a GWAS design [8, 9]. The second possibility is being tested by genome sequencing, with results to date suggesting that rare coding variants will not fully explain the missing heritability [10–14]. By contrast, Park et al. [15] examined GWAS index SNPs across 13 traits and diseases and found that the effect–size distributions suggest the existence of large numbers of disease-associated variants with decreasingly small effects. Similarly, Visscher et al. [1] analyzed multiple GWAS across ethnic groups and found that most of the chromosomal regions that had GWAS peaks in one group also showed associations in others, albeit with differences in allele frequency and linkage disequilibrium (LD) patterns. This suggests that the common-variant signals are likely to be the result of widely distributed causal alleles of relatively high frequency. Findings in other important phenotypes, such as alcoholism, have been consistent with this theme, although sometimes the same gene-containing region can show different peak SNPs in different ethnic groups [16]. Polygenic scores from GWAS summary statistics can be used to model the proportion of overall heritability from common variants [11, 15], and this approach has provided estimates, for example, that about 25% of the heritability of bipolar disorder can be explained by common variants [11]. Likewise, coronary artery disease genetic risk appears to reflect the cumulative effects of multiple common risk alleles, individually of small effect size [17]. Central to the problem of capturing these common variants, many of the interesting signals in well-powered GWAS still do not reach the ~ *p* < 5 × 10^8^ thresholds for genome-wide significance, and are thus suggestive but not strictly accepted. The post-GWAS mapping approaches that we outline in the next sections can be useful for prioritizing these sub-threshold signals for additional scrutiny.

### GWAS and the problem of identifying causal sequence variants

With regard to the second criticism of GWAS, that these studies have not delivered biologically relevant knowledge, there have indeed been frustrations stemming from the fact that about 90% of peak signals from GWAS localize to non-coding sequences [18]. Owing to LD between multiple SNPs in a chromosomal region, GWAS associations typically highlight broad regions spanning 10 to 100 kb of DNA, and the lead SNP is not necessarily the functional source of the association signal. As an example, it took almost 10 years for an obesity locus identified though GWAS to be attributed, at least in part, to the disruption of ARID5B-mediated repression of *IRX3* and *IRX5*, rather than to an alteration of the function of the *FTO* gene in which the original GWAS peak SNP was found [19]. Thus, statistical genetics can point to the vicinity of causal sequence variants but cannot hone in on these variants without using additional types of evidence. This limitation has spurred recent efforts to rank and prioritize candidate variants using functional annotations [20]. Regulatory sequence elements often act in a cell-type-specific manner, so analysis of purified tissues and cell types, including relatively inaccessible ones that are disease-relevant (neurons, pancreatic islet cells, and so on) is crucial for the functional investigation of GWAS variants.

**Fig. 1:**
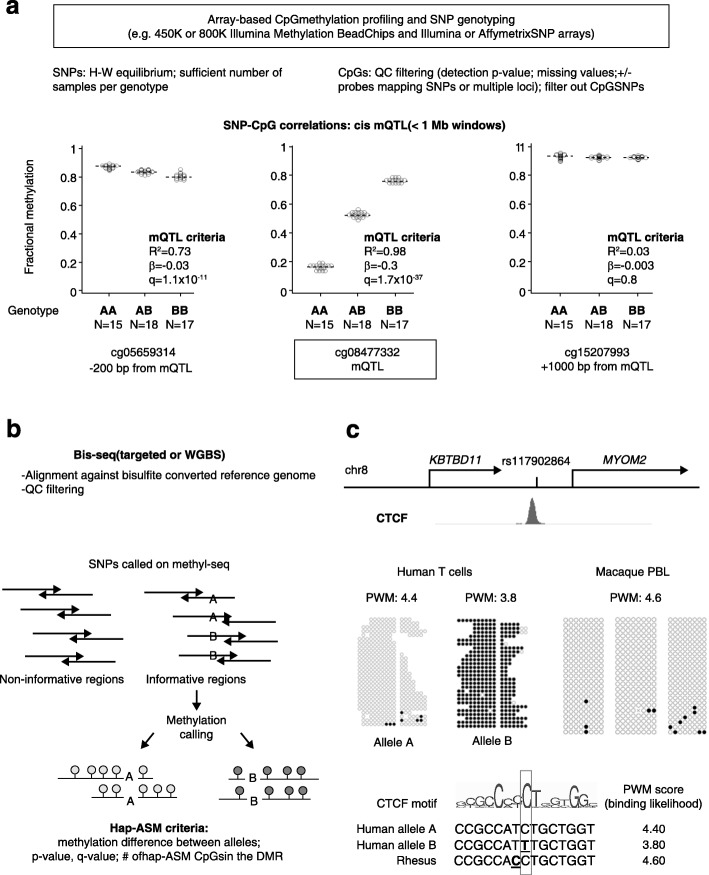
Approaches for mapping mQTLs and hap-ASM DMRs. Haplotype-dependent allelic methylation asymmetry (hap-ASM) can be assessed using two different approaches, methylation quantitative trait locus (mQTL) and hap-ASM analysis. The mQTL approach is based on correlations of (biallelic) net methylation to genotypes across individuals, whereas sequencing-based approaches are based on direct comparisons between alleles in single (heterozygous) individuals. **a** To identify mQTLs, correlations between single nucleotide polymorphism (*SNP*) genotypes and net methylation at nearby CpGs are measured in groups of samples. Methylation and genotyping data are generated in separate assays, which are usually array-based, and correlations are computed using linear regression or Spearman’s rank correlation. The mQTLs are defined using *q* value (false discovery rate [FDR]-corrected *p* value), effect size (β value), and goodness of fit of the linear model (R square). An example of a mQTL in the *S100A* gene cluster [49] is shown. The genotype of the index SNP, rs9330298, correlates with the methylation at cg08477332 by stringent criteria (β > 0.1, R^2^ > 0.5, *q* value <0.05). Lack of correlations between the index SNP and more distant CpGs corresponds to a discrete hap-ASM region spanning approximately 1 kb. **b** Hap-ASM is analyzed directly, using targeted bis-seq or whole genome bisulfite sequencing (*WGBS*) in single individuals. Deep long-read sequencing is desirable to generate reads mapping both CpG sites and common SNPs because the statistical power depends on the number of reads per allele. Alignment is performed against bisulfite-converted reference genomes, which can be done, for example, using Bismark [169], BSMAP [170], or Bison [171]. Alignment against personalized diploid genomes (constructed using additional genotyping data) or SNP-masked reference genomes, can decrease alignment bias toward the reference allele. Quality control (*QC*) filtering is based on Phred score, read length, duplicates, number of mismatches, ambiguous mapping, and number of reads per allele. CpG SNPs can be tagged or filtered out by intersecting CpG and common SNP coordinates. After alignment and quality control of the bis-seq data, SNP calling is performed, for example, using BisSNP [172]. For C/T and G/A SNPs, the distinction between the alternative allele and bisulfite conversion is possible only on one of the DNA strands (the G/A strand). Methylation levels are determined separately for the two alleles, both for individual CpGs and for groups of CpGs in genomic windows, and compared using, for example, Fisher’s exact test or Wilcoxon test, respectively. Both *p* value (and corrected *p* value) and effect size metrics (number of significant CpGs in the DMR and methylation difference across all covered CpGs) are used to define hap-ASM regions. **c** Example of a hap-ASM DMR, located downstream of the *KBTBD11* gene [49]. The hap-ASM region in T cells overlaps a CTCF ChIP-Seq peak. The index SNP (rs117902864) disrupts a canonical CTCF motif as reflected by a lower position weight matrix (PWM) score associated with allele B. This result implicates CTCF allele-specific binding as a mechanism for hap-ASM at this locus. Consistent with this hypothesis, the NHP (Rhesus macaque) sequence differs from the human reference allele (allele A) by one nucleotide (*bold and underlined*) which does not affect the binding affinity, and the observed methylation levels are very low in the macaque blood samples, similar to allele A in the human T cells. *PWM* position weight matrix

When applied to appropriate cells and tissues, the allele-specific mapping approaches that we describe in the next sections can help to extract maximum biological information from GWAS data. These approaches are of two general types: QTL and allele-specific analyses (Fig. 1). In quantitative trait locus (QTL) approaches, the functional effect of a given variant is assessed by correlating the bi-allelic net effect (e.g., expression, methylation) with separately generated genotyping data. Such data are most often array-based, permitting the study of large populations in a cost-efficient manner, but with the technical issues inherent to arrays, such as variations in probe hybridization, batch effects, and limited genomic coverage. In more direct approaches, massively parallel sequencing methods, including bisulfite sequencing (bis-seq) for CpG methylation, are used to assess the allele-specific effects of variants or haplotypes after separating the sequenced DNA fragments by allele. While QTL approaches are based on correlations across individuals, sequencing-based approaches are based on the direct comparison of alleles in single individuals. The advantages of allele-specific approaches are smaller sample size requirements and more complete genomic coverage, but drawbacks can include greater cost per sample and more complex data processing and analysis.

## Post-GWAS mapping methods: eQTLs and ASE

Efforts to extract maximum information from GWAS data can benefit from a multi-pronged approach that uses several mapping strategies to query the functional effects of non-coding sequence variants. Among these methods, the first to be developed utilized eQTLs, that is, SNPs at which the genotype correlates with expression of one or more nearby genes. Mapping of eQTLs within haplotype blocks that are implicated by GWAS can provide links to genes whose genetically regulated expression may be involved in the phenotype [21, 22]. Initial studies were performed on lymphoblastoid cell lines (LCLs), including samples from the Centre d'Etude du Polymorphisme Humain (CEPH)/HapMap projects [23–28]. Microarray data were utilized to probe the relationships between genetic polymorphisms and mRNA expression levels, and the results uncovered a pervasive *cis*-acting influence of SNPs (and thus haplotypes) on gene expression. Schadt et al. [27] estimated the heritability of the gene expression phenotypes in CEPH pedigrees and concluded that about 25% of genes had heritable variation, whereas a study from the Pastinen lab comparing SNPs in cDNAs to paired genomic DNA samples found that about 10% of expressed genes in LCLs show genotype-linked ASE [29]. Stranger et al. [24] showed that both SNPs and, at a lesser frequency, copy number variants (CNVs) are implicated in this phenomenon. Searching for *trans*-acting eQTLs can present computational challenges, but so far it appears that *cis*-acting eQTLs are more common than those that act in *trans* [30, 31].

### Cell type-specific and disease-specific eQTL or ASE mapping

Early on, Pastinen and Hudson [32] pointed out that eQTLs are likely to be cell-type-specific. With more recent studies on T lymphocytes, monocytes, skeletal muscle, liver, brain, and other tissues and cell types, we now have a clear picture of the tissue-specificity and frequencies of eQTLs or ASE. The earlier studies relied on microarray data, whereas the more recent studies have mostly utilized RNA-seq, combined with genomic sequencing or array-based SNP genotyping. In their analysis of human T cells in a small series, Heap et al. [33] found that about 5% of genes showed an allelic expression bias passing their numerical criteria, while in a larger study of total peripheral blood (PBL) samples, Battle et al. [34] detected SNPs that, using their statistical cutoffs, influenced the ASE of over 10,000 genes. A similarly designed study of brain frontal cortex found that approximately 9% of the transcripts showed a genome-wide significant correlation with the genotypes of nearby SNPs [35], and analyses of human monocytes showed that approximately 20% of genes are influenced by eQTLs [30, 36]. The number of loci scored as positive for eQTLs or ASE depends on the stringency of the cutoffs that are used to define a significant allelic bias, and for practical applications, the stronger eQTLs are of most interest. Useful in this regard is a recent large-scale study from the International Human Epigenome Consortium (IHEC), which applied RNA-seq to several immune cell types from approximately 200 individuals and found a greater than two-fold allele-specific bias (strong ASE) in about 3% of transcripts [37].

While eQTLs or ASE can be adequately analyzed using sufficiently powered sets of non-diseased samples, because of differences in allele frequencies in cases versus controls, some eQTLs that are relevant to a given disease are more likely to be discovered if the sample set includes disease cases. The activation state of a given cell type in response to signaling ligands can also matter: Fairfax et al. [38] found that in vitro stimulation of primary human monocytes can abrogate and induce specific eQTLs, and Peters et al. [39] performed eQTL mapping in five primary immune cell types from patients with inflammatory diseases and found a small but interesting subgroup of eQTLs that were present only in those with active disease. These technical considerations are also important in designing studies of mQTLs and hap-ASM, which we discuss below.

### Co-localization of ***eQTLs*** and GWAS peaks

**Fig. 2:**
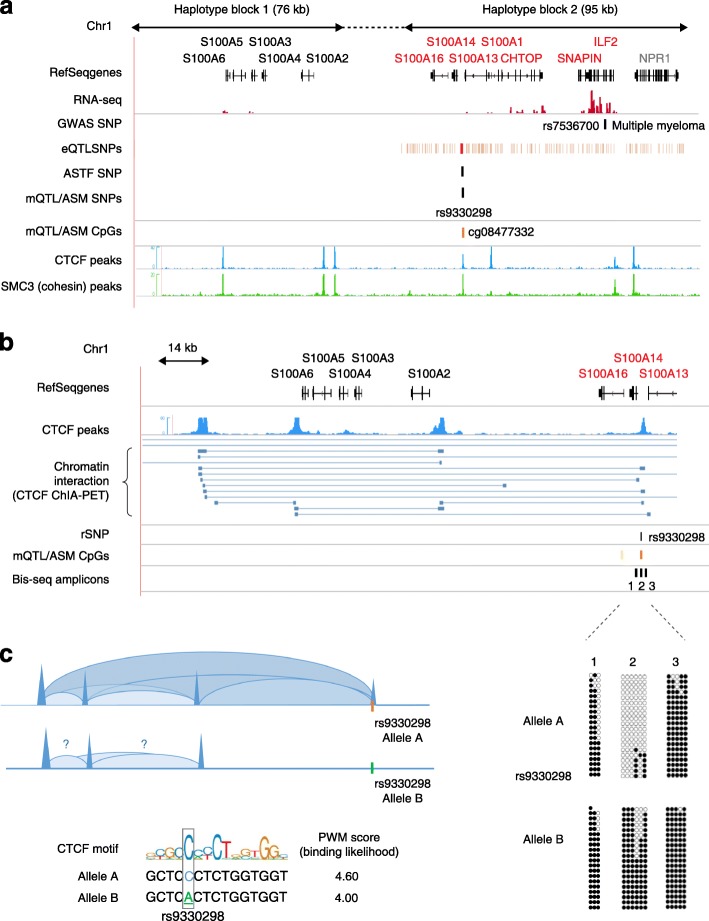
Integrative “post-GWAS” mapping of allele-specific marks for identifying disease-associated regulatory sequence variants. Genome-wide association studies (*GWAS*) typically implicate a haplotype block spanning tens to hundreds of kilobases, with resolution limited by the fact that all single nucleotide polymorphisms (*SNP*s) that are in strong linkage disequilibrium (LD) with the index SNP will show a similar disease association. A combination of post-GWAS modalities using maps of allele-specific marks can help to localize the causal genes and the underlying regulatory sequences. **a** The S100A*-ILF2 region exemplifies this approach. The map shows the index SNPs for expression quantitative trait loci (*eQTL*s), methylation quantitative trait loci (*mQTL*s), haplotype-dependent allele-specific DNA methylation (*hap-ASM*), and allele-specific transcription factors (*ASTF*). The suggestive (sub-threshold) GWAS signal for multiple myeloma susceptibility (rs7536700, *p* = 4 × 10^−6^) tags a haplotype block of 95 kb, which was defined using 1000 Genome data [186] with an algorithm that emphasizes D-prime values [187, 188]. The GWAS SNP overlaps no known regulatory element or transcription factor (TF) binding site. Numerous *cis*-eQTL SNPs correlating with several genes within 1 MB have been identified in this haplotype block (eQTL-tagged genes indicated in *red*), so identifying the causal regulatory SNP(s) is not possible solely from eQTL data. However, several SNPs in the block identify mQTLs, all correlating with the same CpG site, cg08477332. Fine mapping using targeted bis-seq [49] confirmed a discrete hap-ASM differentially methylated region (DMR; *orange*) spanning ~1 kb. The hap-ASM index SNP rs9330298 is in strong LD with rs7536700 (D′ = 1), is the closest SNP to the DMR, and is an eQTL correlating with *S100A13* expression. In addition, this DMR coincides with a CTCF peak that shows allele-specific binding in chromatin immunoprecipitation-sequencing (ChIP-Seq) data, nominating the disruption of CTCF binding by rs9330298 as a candidate mechanism underlying susceptibility to multiple myeloma, either by direct effects in B cells or via effects on immune surveillance by T cells. The eQTL and ASTF data are from the Genotype-Tissue Expression project (GTEx) and alleleDB, respectively [47, 180]. RNA-seq data in GM12878 cell lines were downloaded from ENCODE. The mQTL and hap-ASM data are from [49], and the CTCF ChIP-seq data (GM12878 LCL) from ENCODE. The *dashed line* represents a genomic region lacking defined LD structure. **b** Map showing three-dimensional chromatin interactions in the S100A* gene cluster. The hap-ASM region coincides with a CTCF-mediated chromatin anchor site, as suggested by chromatin interaction analysis by paired-end tag sequencing (*ChIA-PET*) data (K562 cell line) [122]. This evidence suggests that disruption of the CTCF-binding site by the candidate regulatory SNP (*rSNP*), rs9330298, might abrogate the formation of one or more chromatin loops. **c** Bis-seq (*closed circles*, methylated CpGs; *open circles*, unmethylated CpGs) confirms that the hap-ASM DMR overlaps a CTCF-binding site (amplicon 2) and the lower position weight matrix (*PWM*) score for allele B of rs9330298 predicts allele-specific disruption of CTCF binding, consistent with the allele-specific binding seen in the ChIP-seq data. The disruption of this CTCF-mediated chromatin anchor site could account for eQTLs in this region, where the *S100A* cluster genes are no longer insulated from the active enhancers of neighboring genes, such as *ILF2* or *CHTOP*, which have higher expression levels in blood

How effective has eQTL/ASE mapping been in extracting biological information from GWAS data? As found by Nica et al. [40] in LCLs and substantiated by Zhang et al. [31] in their meta-analysis of multiple eQTL studies which they overlapped with human GWAS, eQTLs are enriched near positive GWAS statistical signals. In an early example of the use of eQTLs as a post-GWAS modality, Zhong et al. [41] focused on type 2 diabetes mellitus (T2D) and integrated GWAS data with eQTLs from liver and fat, which led them to a collection of GWAS peaks (index SNPs) and associated eQTLs that were enriched for genes acting in relevant signaling pathways. An important limitation in the identification of disease-associated genes is that *cis*-eQTLs occur quite frequently, leading to very dense maps, as shown for a typical genomic region in Fig. 2. Consequently, it remains challenging to identify the specific functional SNPs by this method [42], and statistical approaches are required to test formally for co-localization of an eQTL and a disease-associated SNP [43, 44]. Importantly for this type of application, eQTL and GWAS results have now been made available as community resources (Box 1). These user-friendly databases include the National Heart, Lung and Blood Institute (NHLBI)-GRASP v2.0 (https://grasp.nhlbi.nih.gov/Overview.aspx), which contains approximately 8.9 million SNP–phenotype associations from more than 2000 GWAS, with annotation sources including eQTLs from liver, adipose tissues, various brain tissues, and blood lineage cells, including PBL, lymphocytes, monocytes, osteoblasts, fibroblasts, and LCLs, as well as growing collections of mQTLs, protein QTLs, and microRNA QTLs [31, 45, 46]. The Genotype-Tissue Expression (GTEx) project is another important database that contains information for both eQTLs/ASE and allele-specific transcription factor (ASTF) binding from multiple human tissues [47].

## Post-GWAS mapping methods: mQTLs and ASM

Because there are typically many common SNPs in LD within a haplotype block, maps of eQTLs can suggest which genes are implicated by a given GWAS peak, but cannot pinpoint the underlying DNA sequence variants. To hone in on a causal regulatory SNP (rSNP) variant, additional types of evidence are needed—preferably from mapping methods that score physical (and thus potentially biologically functional) differences between two alleles. One approach stems for the discovery of mQTLs and hap-ASM. The terms mQTL (strictly speaking, *cis*-mQTL) and hap-ASM both describe the same class of allelic asymmetry, in which the DNA methylation on each allele depends on the local DNA sequence (i.e., the haplotype). However, as shown in Fig. 1, they are mapped by different strategies: mQTLs by searching for correlations of net methylation at individual CpGs with the genotypes of nearby SNPs in large sets of samples, and ASM by directly measuring differences in the methylation levels of CpGs on the two different alleles in individual heterozygous DNA samples, using bis-seq. Although the methods for their discovery differ, the physical basis of mQTL and hap-ASM is identical, so when assessed by appropriate assays, all bona fide mQTLs should turn out to correspond to allele-specific differentially methylated regions (DMRs) and vice versa.

Examples of genome-wide studies of ASM and mQTLs, along with the profiling platforms, cell types, and tissues examined, and summaries of the main findings are listed in Tables 1 and 2. The first genome-wide scans for ASM were done by the methylation-sensitive SNP array (MSNP) method. In this approach, genomic DNAs are pre-digested with methylation-sensitive restriction enzyme(s) as well as standard non-methylation-sensitive enzymes, and duplicate samples are digested only with the non-methylation-sensitive enzymes. This step is followed by probe synthesis and hybridization to SNP chips, and the readouts are allele-specific hybridization intensities. In our early MSNP study of several normal human tissues, we found many examples of ASM, which mostly showed strong correlations with local SNP genotypes, indicating *cis*-regulation [48]. Other laboratories applied MSNP to other types of cells and tissues and obtained similar findings of widespread *cis*-regulated ASM (examples in Table 1). Analogously to the situation for eQTLs, the ASM phenomenon shows tissue-specificity, with some loci having pan-tissue ASM and others having strong ASM only in one tissue or cell type.

For SNP-tagged loci in which ASM is detected in groups of heterozygous individuals, binomial or Fisher exact tests can be used to ask whether the relatively hypermethylated allele tracks with one SNP genotype, a sign of hap-ASM. Importantly, for loci in which a positive but not absolute correlation is seen with the closest “ASM index SNP”, extended genotyping over multiple SNPs can sometimes reveal a perfect association of the hypermethylated allele with a specific haplotype [49]. By contrast, genomic imprinting, which affects about 100 human genes [50], is associated with ASM that is parent-of-origin-dependent, not haplotype-dependent. Therefore, in all studies of ASM, it is important to identify known imprinted loci and to exclude them from downstream analyses. The distinction can also be made empirically using trios of maternal, paternal, and offspring samples, asking whether the hypermethylation is consistently found on an allele marked by the same SNP genotype or, alternatively, whether it is random with respect to SNP genotypes across the series but tracks reliably with the maternally or paternally transmitted allele [48, 49]. In fact, MSNP and reduced representation bis-seq (RRBS) approaches uncovered not only hap-ASM loci, but also novel examples of imprinted genes [51, 52]. An interesting and unexpected interaction between imprinting and hap-ASM is highlighted by our recent observation that the *ZFP57* gene, which codes for a transcription factor that functions as a *trans*-acting modifier of DNA methylation at certain imprinted loci, is in turn regulated by hap-ASM [49].

The number of scans for ASM using Agilent Methyl-seq and other genome-wide bis-seq methods has grown with the increasing availability of those technologies, and is matched by the proliferation of array-based mQTL mapping studies (Tables 1 and 2) [37, 53–58]. An advantage of directly mapping ASM is that, unlike mQTL analyses, which require large numbers of samples, bis-seq for ASM can be informative in single heterozygous individuals. Shoemaker et al. [59] used padlock probes with bis-seq in a panel of 16 human cell lines including induced pluripotent stem cells and, using lenient statistical criteria for allelic bias, concluded that ASM is present in the vicinity of around 20% of heterozygous SNPs. Li et al. [60] reported genome-wide bis-seq data from one sample of human peripheral blood mononuclear cells and found 599 haploid DMRs covering 287 genes. We recently applied array-based methylation and SNP genotyping and Agilent Methyl-seq with a mean depth of coverage ranging from 50× to 94× as complementary approaches and, in a panel of tissues, identified 795 strong hap-ASM DMRs that were detectable in one or more tissue types [49] (examples in Table 3). Encouragingly, ASM data from independent laboratories are converging: Cheung et al. [28] mapped ASM and mQTLs using MethylC-Capture Sequencing (MCC-seq) in 869 samples, including whole blood, monocytes, T cells, muscle, and visceral adipose tissue, and whole genome bisulfite sequencing (WGBS) for a subset of 41 samples, with a mean coverage ranging from 13× to 24× for MCC-seq and 8× to 22× for WGBS. After pooling bis-seq reads across individuals according to genotype and cell type, they identified a large number of ASM CpGs in one or more cell types, which largely encompass the group of ASM CpGs from our study.

**Table 3:** Examples of hap-ASM DMRs associated with eQTLs and GWAS peaks

Hap-ASM DMR index SNP in haplotype block 5	Regulome-DB score	Genes in 150-kb window	Genes with *cis*-eQTLs in haplotype block	GWAS index SNPs and disease associations in haplotype block
rs9535274	1b	*RCBTB1*; *ARL11*; *EBPL*	*RCBTB1*; *ARL11*; *EBPL*	rs9568281: multiple sclerosis
rs9330298	2a	S100A* cluster; *CHTOP*; *SNAPIN*; *ILF2*; *NPR1*; *MIR8083*	*S100A1*, *S100A13*	rs7536700: multiple myeloma^b^
rs12789117	5	*JAM3*; *NCAPD3*; *VPS26B*	*JAM3*; *NCAPD3*; *VPS26B*	rs1267813: schizophrenia rs11223731: memory performance rs1031381: neuropsychological test rs478881: fasting blood insulin
rs2517646	1b	TRIM* cluster	*TRIM10*	rs2523989: type I diabetes rs2021722: schizophrenia, bipolar disorder
rs994379	1f	HIST1H* cluster; *BTN3A2*	HIST1H* cluster; *BTN3A2*	rs61747867: schizophrenia^b^
rs8176749	5	*OBP2B*; *ABO*; *SURF6*	*ABO*, *SURF6*	rs633862: ovarian cancer^b^ rs495828: thromboembolism rs8176722, rs8176719: malaria rs579459: coronary heart disease
rs861855	1b	*UBE2L3*; *YDJC*; *CCDC116*; *SDF2L1*; *MIR301B*; *MIR130B*; *PPIL2*; *YPEL1*	*CCDC116*; *YDJC*; *UBE2L3*	rs181359, rs2256609: Crohn’s disease rs131654: systemic lupus erythematosus rs2266961: inflammatory bowel disorder rs2256609: Crohn’s disease rs4821116: hepatitis B infection rs2298428: celiac disease^b^
rs1627982	4	*HLA-H*; *HCG4B*; *HLA-A*; *HCG9*; *ZNRD1-AS1*	*HLA-A*; *HCG9*; *HCG4*; *ZNRD1*; *HLA-H*	rs2523809: serum IgE rs2860580: nasopharyngeal cancer rs2524005: schizophrenia, bipolar disorder rs189370103: smoking behavior
rs62396301^a^	4	*UNC5CL*; *TSPO2*; *APOBEC2*; *OARD1*; *NFYA*; *ADCY10P1*; *TREML1*; *TREM2*	*NFYA*; *APOBEC2*	rs75932628: Alzheimer’s disease rs2294693: gastric cancer^b^

The hap-ASM data are from our published study [49], with confirmation by additional unpublished Methyl-seq data (CD and BT; unpublished data). Of these nine loci, six were also covered and found to have ASM or mQTLs in one or more cell types by Cheung et al. [53]. Regulome-DB scores for the hap-ASM index SNPs are from RegulomeDB (http://www.regulomedb.org/). The scores ranged from 1a to 6, with 1 assigned to putative regulatory SNPs with the highest level of confidence, supported by multiple data types, including eQTLs, TF binding, TF motifs, DNAse footprints, and DNAse hypersensitivity peaks [20]. *Cis*-eQTLs were downloaded from National Heart, Lung and Blood Institute (NHLBI)-GRASP Build 2.0 [46], only genes with eQTL *p* value <10^−05^ are listed. Haplotype blocks were defined using 1000 Genomes project (phase 3) [182] and PLINK (Gabriel’s approach) data [183, 184]. The S100A* cluster includes: *S100A4*; *S100A3*; *S100A2*; *S100A16*; *S100A14*; *S100A13*; and *S100A1*. The HIST1H* cluster includes: *HIST1H1D*; *HIST1H4F*; *HIST1H4G*; *HIST1H3F*; *HIST1H2BH*; *HIST1H3G*; *HIST1H2BI*; and *HIST1H4H*. The TRIM* cluster includes: *TRIM10*; *TRIM15*; and *TRIM26*. Multiple eQTLs have been identified in the haplotype blocks; in the eight first examples, at least one of the eQTLs was also an ASM index SNP, suggesting that these SNPs are regulatory SNPs

^a^Index eQTL reported in NHLBI-GRASP is rs6926079, in the same haplotype block as rs62396301 (R^2^ = 0.975, D′ = 1)

^b^Sub-threshold GWAS peaks (5 × 10^–6^ < *p* value < 5 × 10^–8^)

Although the number of loci identified depends on sample sizes, depths of coverage for ASM analysis, and numerical cut-offs and *p* values, the yield of mQTL/ASM loci in these studies suggests that approximately 10% of all human genes are associated with strong hap-ASM DMRs. Among the consistently noted features of hap-ASM is its tendency to be located outside of CpG islands and further away from genes [61, 62]. This feature may indicate that ASM events occur in regions that are subject to less stringent selective constraints in evolution. Nevertheless, a substantial minority of hap-ASM DMRs are located in crucial regulatory sequences, including enhancers and insulators [49, 51, 53, 61, 63], and are therefore likely to have important effects on gene expression.

Most studies on *cis*-acting genetic effects in human cells and tissues have focused on epigenome-wide statistics, which are crucial for testing mechanistic hypotheses. Presenting the data in this way can, however, make it difficult to appreciate the patterns and strength of allele-specific epigenetic asymmetries at specific loci. To fill this gap, we have taken pains to illustrate bis-seq of individual loci with ASM, using SNPs in the sequence reads to separate the two alleles [49, 51]. Examples of diagrams of ASM from this procedure (Figs. 1 and 2) show that the allelic bias in CpG methylation can be quite strong [48, 49, 51]. Fine-mapping of ASM DMRs using targeted bis-seq can define the boundaries of these regions, which is a crucial step in testing the candidate biological mechanisms that we discuss in the next section.

## *Cis*-acting mechanisms: involvement of CCCTC-binding factor (CTCF) and transcription factors

The challenge of understanding the mechanisms that lead to mQTLs and hap-ASM is related to the more general question of how CpG methylation patterns are established in mammalian cells. In addition to the involvement of epigenetic “reader and writer” enzymes, multiple lines of evidence are starting to imply roles for sequence-specific DNA-binding proteins, including classic transcription factors (TFs) and insulator binding proteins that regulate three-dimensional (3D) chromatin architecture. The binding of some proteins to DNA protects their binding sites in the DNA from CpG methylation; such proteins include zinc-finger CxxC-domain-containing proteins, such as CFP1 and KDM2A, the insulator binding factor CTCF, which anchors chromatin loops and thereby regulates promoter–enhancer interactions [51, 64–69], and TFs including ETS-family DNA-binding proteins and SP1. Some of the implicated proteins show methylation-sensitive DNA binding [70–73], but another working hypothesis is that simple site occupancy may be sufficient to exclude methylation from that site.

### Cross-talk between DNA methylation and sequence-specific binding proteins

There may be a “chicken or egg” problem in determining whether binding site occupancy or site methylation status is primary, but the fact is that binding sites tend to be hypomethylated when occupied. Stadler et al. [74] profiled genome-wide patterns of CTCF binding sites in mouse embryonic stem cells (ES) and ES-derived neuron progenitors and found an average CpG methylation of 20% in CTCF-binding sites, with increasing methylation adjacent to these sites, leading to “methylation well” patterns. Xu et al. [75] extended this principle in a survey involving multiple cell lines, TFs, and methylation types, which revealed intimate relationships between occupancies of TFBS and methylation levels in and around these sites. Likewise, chromatin-immunoprecipitation (ChIP) against CTCF in ES, followed by bis-seq of the immunoprecipitated DNA, led to the observation that the frequency of CTCF binding correlates with the likelihood of a demethylated state [76]. Our data from Agilent Methyl-seq of T cells and brain DNAs, aligned with ENCODE CTCF ChIP sequencing (ChIP-Seq), are in line with these findings [49].

Conversely, a group of zinc-finger TFs, including the BTB/POZ family proteins KAISO, ZTB4, and ZBTB38, as well as the Krüppel-associated box (KRAB)-domain TF family member ZFP57, all recognize methylated CpGs within DNA sequence motifs and can act as repressors by perpetuating local CpG hypermethylation [77]. A protein microarray-based approach for surveying purified human TFs revealed numerous examples, typified by the Krüppel-like zinc-finger domain protein KLF4, which showed methylated CpG-dependent DNA-binding activities [78]. Very recently, Yin et al. [79] showed that most major classes of TFs, including bHLH, bZIP, and ETS, bind preferentially to unmethylated DNA, whereas other TFs, such as homeodomain, POU, and NFAT, bind preferentially to methylated DNA. Last, methyltransferase enzymes themselves can show some DNA-sequence preferences [80, 81], and members of the methyl-binding proteins family (e.g., MeCP2 and MBD2), while lacking sequence-specificity, participate in protein complexes that bind highly methylated CpG-rich sequences and can help to maintain repressive chromatin [82].

### Allele-specific TFBS occupancy as a mechanism for ASM

Early on, we proposed that ASTF binding site occupancy (sometimes abbreviated as ASB, for allele-specific binding) resulting from the presence of sequence variants in enhancer and insulator elements could lead to ASM [83]. In fact, ASTF was documented as a pervasive phenomenon in human cells at around the same time that hap-ASM was first being characterized: allele-specific ChIP-on-chip assays using antibodies to RNA polymerase II and post-translationally modified forms of histone H3, together with SNP genotyping, revealed evidence of widespread allele-specific chromatin states [84–86]. With the advent of ChIP-seq, experiments with denser genomic coverage have confirmed these findings, and have added assays for the binding of specific TFs that highlighted ASTF for CTCF, NF-kappaB, ETS1, ELF1, PAX5 and RUNX proteins, among others [87–93]. In a parallel line of work, Butter et al. [94] used SILAC (Stable Isotope Labeling by Amino acids in Cell culture), in which double-stranded oligonucleotides of the two alleles for many TFBSs were incubated with either light or heavy isotopically labeled nuclear extracts, and subsequently mass spectrometry to detect altered TF binding to the SNP-containing sequences. Using this method, they found allele-specific binding of the TFs RUNX1, LEF1, CREB, and TFAP4 to polymorphic SNP-containing TFBSs. AlleleDB (http://alleledb.gersteinlab.org/) is a useful public resource for querying and analyzing ASTF [47]. Although the current database is skewed toward cell lines, it is expected to include multiple primary cell types in the near future.

In testing ASTF as a mechanism underlying hap-ASM, it is crucial to know which TFs bind to each ASM DMR, and hence it is necessary to determine the sizes and boundaries of these DMRs. An initial fine-mapping study of several strong examples of hap-ASM DMRs showed allelic asymmetries in methylation over multiple CpG dinucleotides, with discrete DMRs of 1–2 kb in size that in some cases showed a precise overlap with CTCF ChIP-seq peaks [51]. As a next step in testing mechanisms, we and others carried out bioinformatic enrichment analyses of epigenome-wide ASM and mQTL mapping data, in which the frequencies of specific sequence motifs, ChIP-seq peaks, and chromatin states in and around the identified ASM DMRs and mQTLs are compared with the overall representation of such motifs and states in the informative fraction of the genome. In their study of LCLs, Banovich et al. [95] found that SNPs in TFBSs that change the predicted binding of cognate TFs are enriched for associations with nearby mQTLs. They used available DNase-seq data to infer sites that are putatively bound by TFs, and then identified SNPs disrupting these putative binding sites. On the basis of known binding motifs, they calculated a position weight matrix (PWM) score for each allele and found that alleles with lower predicted TF-binding affinity (lower PWM scores) tend to be associated with increased DNA methylation in 1-kb windows centered on the binding sites. These data suggested that TFBS occupancies by CTCF, PAX9, ESE1, STAT5, and ZNF274 play a role in shaping CpG methylation patterns in LCLs. In our recent Methyl-seq study, we found that hap-ASM DMRs are enriched in strong CTCF-binding peaks that are restricted to one or multiple cell types, but not in “constitutive” CTCF peaks that are identified in almost all cell types [49]. We found significant enrichment in polymorphic but not invariant CTCF motifs, supporting allele-specific CTCF binding as a mechanism that underlies a subset of hap-ASM loci [49]. To assess the involvement of classic TFs, we overlapped our ASM and mQTL data with all TF canonical motif occurrences in the ENCODE data and found that hap-ASM loci are significantly enriched in polymorphic TF-binding motifs, supporting a role for allele-specific TF-binding site occupancies in creating and/or propagating the ASM [49].

## Cross-species comparisons for testing mechanisms of ASM

Cross-species designs comparing methylomes in humans and other animals, such as mice and non-human primates (NHPs), are proving to be informative. Genetically influenced ASM has been demonstrated in mouse crosses [96], which offers the possibility of doing manipulative genetic experiments. However, a key advantage of comparing humans to NHPs, rather than mice, is that chimpanzees and monkeys are “almost human”, both in their anatomy and physiology and in their genomes. This fact should be especially important for traits related to brain function. Although there are many insertion-deletion polymorphisms and structural rearrangements in each primate species, yielding an overall sequence divergence of about 5%, many orthologous portions of the genomes differ by only approximately 1.5% [97–99]. This situation allows comparison of CpG methylation patterns in and around regulatory sequences that are either identical in humans and the NHP species or differ by only one or two nucleotide substitutions. Thus, by expanding the range of evaluable alleles beyond those found in human populations, the NHP methylomes add power to studies that seek to use maps of ASM to hone in on functional variants in TFBS.

Kasowski et al. [87] compared PolII binding in human LCLs and a chimpanzee blood sample using ChIP-seq and found divergence at a substantial subset of orthologous genes between the two species. Similarly, methylome mapping studies have highlighted genes that are perfectly conserved in their protein-coding sequences, yet show significant differences in CpG methylation levels in their regulatory sequences between humans and chimpanzees [100, 101]. Relevant to hap-ASM, in our recent work, we used cross-species comparisons of DNA sequences and methylation patterns to test variation in CTCF-binding sites as an underlying mechanism. We carried out targeted bis-seq in PBL and liver from macaque monkeys at five ASM loci orthologous to human loci with CTCF motifs, selecting these DMRs so that the macaque sequences diverged from the human sequences at only one or two critical base pairs in these motifs. As shown for an example in Fig. 1, the results for each of these DMRs confirmed the expected negative correlation between their CpG methylation levels and CTCF-binding likelihood, as indicated by the PWM scores, when comparing the human and monkey alleles [49]. Such cross-species studies can now be performed using combinations of whole genome and whole methylome sequencing, which are expected to yield additional important clues to functional DNA regulatory variants and the TFs that recognize them.

## Some ASM DMRs remain mechanistically unexplained

**Fig. 3:**
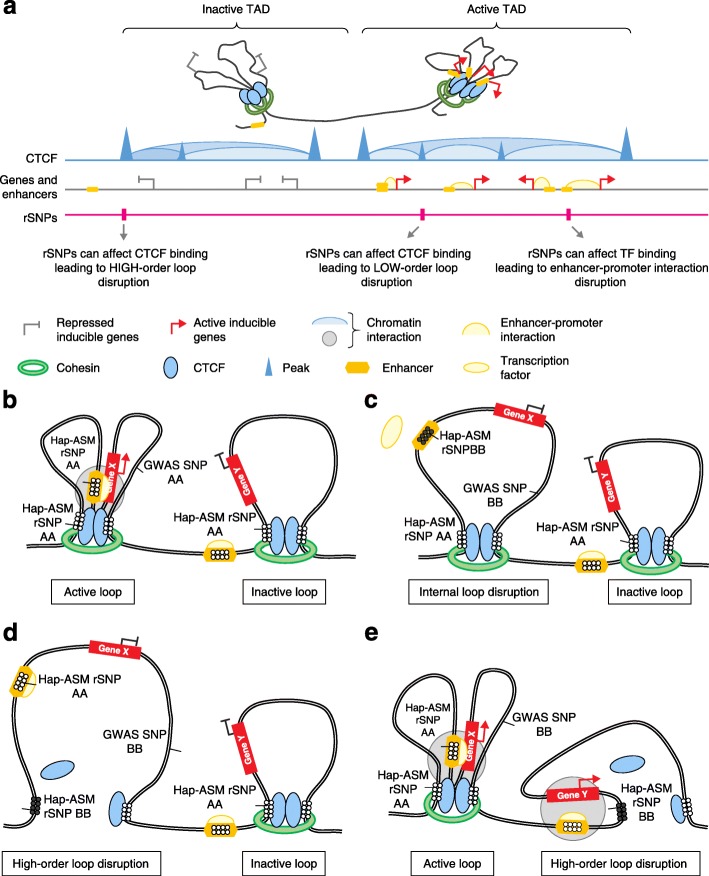
*Cis*-acting genetic–epigenetic interactions can lead to inter-individual differences in DNA looping, gene expression, and disease susceptibility. Simplified representations of three-dimensional chromatin structure in haplotype blocks containing genome wide association study (*GWAS*) peaks, highlighting the potential effects of regulatory sequence variants (*rSNP*s) on DNA methylation, interactions between regulatory elements (insulators, enhancers and promoters), topologically associating domain (*TAD*) structures, gene expression, and disease susceptibility. **a** CTCF-mediated chromatin looping leading to formation of “active” and “inactive” TADs. Chromatin interaction analysis by paired-end tag sequencing (ChIA-PET) and Hi-C have mapped chromatin interactions and have identified TADs as large-scale chromatin structures, with CTCF or cohesin enriched at the TAD boundaries [103]. The chromatin loops promote intra-domain interactions between regulatory elements, such as enhancers and gene promoters (which induce gene expression), while preventing inter-domain contacts in order to minimize promiscuous gene expression. In this model, regulatory variants at TAD boundaries or intra-domain contacts (sub-TAD boundaries) can induce high- or low-order chromatin configuration changes that disrupt the insulated neighborhoods formed by the looping, thereby causing either the abolition of enhancer–promoter interactions (in active TADs) or the formation of ectopic enhancer–promoter interactions (in inactive TADs). Additionally, regulatory variants at active transcription factor (TF)-bound enhancers can directly affect enhancer–promoter interactions. Variants that affect the integrity of TAD structures and chromatin interactions are more likely to have functional effects and to be rSNPs, which can sometimes lead to disease susceptibility. **b** Chromatin looping leads to active or inactive insulated chromatin neighborhoods, which can vary between individuals because of haplotype-dependent allele-specific DNA methylation (*hap-ASM*) rSNPs and can therefore influence DNA methylation patterns and disease susceptibility. In this genomic configuration (AA alleles at the enhancer SNP of gene X, AA alleles at the CTCF-binding site SNP of the gene-X-containing loop, and AA alleles at the CTCF-binding site SNP of the gene-Y-containing loop), both of the TAD anchor sites have a high affinity for CTCF. In the chromatin loop associated with gene X, the formation of the loop brings the enhancer and promoter into close proximity. The active enhancer is bound by TFs and RNA polymerase interacts with the gene X promoter to induce transcription [122, 189]. Conversely, the chromatin loop containing gene Y enforces gene silencing by isolating the promoter away from neighboring enhancers. CTCF and TF occupancy is associated with low methylation at the TAD anchor sites and in enhancer sequences, expression of gene X, silencing of gene Y, and no disease susceptibility. **c** In this configuration (BB at the enhancer SNP of gene X, AA at the CTCF-binding site SNP of the gene-X-containing loop, and AA at the CTCF-binding site SNP of the gene-Y-containing loop), the anchor sites bind CTCF with high affinity. Although the CTCF-anchored loops are not altered, the rSNP at the enhancer of gene X disrupts the binding of the TF and RNAPII complex, resulting in a high methylation level at the enhancer and gene silencing. In this scenario, the silencing of gene X leads to disease susceptibility, associated with the GWAS index SNP allele BB, which is in linkage disequilibrium (LD) with the functional rSNP allele BB at the enhancer of gene X. **d** In this configuration (AA at the enhancer SNP of gene X, BB at the CTCF-binding site SNP of the gene-X-containing loop, and AA at the CTCF-binding site SNP of the gene-Y-containing loop), allele BB at the CTCF-dependent TAD anchor site associated with gene X leads to a low affinity for CTCF. The loss of CTCF binding disrupts the higher-order chromatin loop, and the promoter–enhancer interaction of gene X is no longer facilitated, although TF binding is not altered at the enhancer. **e** In this configuration (AA at the enhancer SNP of gene X, AA at the CTCF-binding site SNP of the gene-X-containing loop, BB at the CTCF-binding site SNP of the gene-Y-containing loop), allele BB at the CTCF-mediated TAD anchor site of the gene-Y-containing loop has a low affinity for CTCF. The loss of CTCF binding disrupts the chromatin loop, such that the promoter of gene Y is no longer isolated from the active enhancer of the neighboring expressed gene, which induces an ectopic enhancer–promoter interaction. This loss of CTCF occupancy is associated with a high methylation level at one of the anchor sites of gene-Y-containing TAD, and expression of gene Y. In this scenario, the expression of gene Y leads to a disease phenotype associated with the GWAS peak SNP allele BB, which is in LD with the causal rSNP allele BB at the CTCF-binding site

Despite this progress in explaining some examples of hap-ASM, a substantial subset of hap-ASM loci are not accounted for by SNPs in known TFBS or CTCF sites ([49] and CD and BT unpublished data). The same is true for other allele-specific chromatin marks. Farh et al. [102] carried out a study of human monocytes, B cells, and resting and stimulated T cell subsets utilizing SNP genotyping combined with genome-wide profiles of histone modifications, RNA-seq, and additional chromatin and TFBS annotations. They found that genetic polymorphisms underlying GWAS peaks for autoimmune disorders tend to occur near TFBS for master regulators of immune differentiation and stimulus-dependent gene activation, but that only 10–20% of presumptive causal genetic variants mapped within recognizable TFBS motifs. One scenario that might explain the “epigenomic dark matter” is long-range chromatin looping. The 3D looping of chromatin into large and complex topologically associating domains (TADs), with loops anchored by CTCF or cohesin complexes [65, 103], is so extensive that a crucial genetically polymorphic CTCF- or TF-binding site may lie at a large linear distance (several megabases or more) from an ASM DMR. Thus, there would be no apparent local explanation for the ASM, even though the binding sites are in fact brought into close proximity to the DMR by chromatin looping (Figs. 2 and 3). Another possibility is a role for long non-coding RNAs (lncRNAs), which are also involved in chromatin looping and transcriptional regulation over long linear distances, via their roles in tethering key protein complexes that regulate epigenetic states. Future experiments that seek to connect hap-ASM to long-range chromatin architecture may eventually explain the epigenomic dark matter.

## Mapping allele-specific epigenetic marks for identifying disease-associated regulatory sequences

Mapping of mQTLs and hap-ASM can be useful for nominating specific polymorphic regulatory DNA sequences as candidates that can account for statistical signals from GWAS. The logic here is that a bona fide regulatory DNA sequence can declare its presence by conferring a physical asymmetry between the two alleles in heterozygotes. Thus, when an SNP association for a given disease or trait is located near an ASM DMR, within the same haplotype block, that signal may be driven, at least in part, by polymorphic regulatory DNA sequences in the DMR [104–107]. In this regard, mQTL/hap-ASM mapping, and related approaches such as the mapping of ASTF, allele-specific histone modifications, and allele-specific chromatin accessibility, are complementary to and non-redundant with eQTL mapping.

As illustrated by the example of the S100A* gene cluster in Fig. 2, and diagrammatically for a generic chromosome region in Fig. 3, some haplotype blocks with GWAS peaks also contain multiple eQTLs and mQTLs. As mentioned above, eQTLs can point to relevant genes, but not necessarily to the relevant DNA sequence elements. Some studies have found only a small overlap (approximately 10%) between the SNPs that tag *cis*-acting mQTLs and eQTLs, which is another rationale for carrying out both types of mapping [49, 108–114]. Dermitzakis and colleagues [115] found that DNA methylation sites that are associated with expression levels are enriched in enhancers, gene bodies, and CpG island shores, but not in promoter regions. These findings are consistent with the fact that eQTLs tend to be found in promoter regions, whereas mQTLs and hap-ASM DMRs tend to occur in non-promoter regulatory sequences such as enhancers and insulators. Despite the relatively low frequency of precise physical overlap, there are many instances in which mQTLs and hap-ASM DMRs map within the same haplotype blocks as eQTLs, and these situations can be informative for understanding disease associations, which may reflect the combined effects of more than one polymorphic regulatory element. With these considerations in mind, an increasing number of recent studies, including ours, have started to catalogue ASM DMRs and mQTLs near GWAS peaks [48, 49, 59] or dictated by GWAS SNPs [37, 116]. Selected examples in which a hap-ASM DMR or mQTL and a GWAS peak are found in a single haplotype block are listed in Table 3.

Other types of allele-specific marks, such as allele-specific histone modifications, have been used for this same purpose [102], and maps of allele-specific chromatin accessibility, scored by the Assay for Transposase-Accessible Chromatin (ATAC) with the high-throughput ATAC-sequencing (ATAC-seq) method, are also starting to be produced [117, 118]. In their study, Scott et al. [118] used RNA-seq plus SNP genotyping to analyze skeletal muscle biopsies from 271 individuals. They integrated the eQTL data with transcriptional regulatory data, including ATAC-seq data, in diverse tissues and found that the tissue-specific regulatory architecture of skeletal muscle is enriched in muscle-specific enhancers, including some that overlap T2D GWAS variants. This finding is biologically relevant since glucose disposal in skeletal muscle is impaired in insulin-resistant states [119]. In addition, FAIRE-seq (formaldehyde-assisted isolation of regulatory elements sequencing) and FAIRE-enriched genotyping are being pursued for identifying allele-specific chromatin accessibility [120, 121]. Another allele-specific mark is allele-specific chromatin topology: using ChIA-PET (chromatin interaction analysis by paired-end tag sequencing) in different cell lines, Tang et al. [122] demonstrated that ASTF of CTCF at TAD anchor sites was associated with allele-specific chromatin interaction and looping, as well as with ASE in lymphoblastoid cells and several cancer cell lines. Among the 32 SNPs disrupting a CTCF motif, they found eight SNPs in LD with GWAS SNPs, supporting allele-specific chromatin topology as a mechanism for disease susceptibility. Follow-up studies will be necessary to extend this result to relevant normal primary cell types.

The overall conclusion is that a multi-modal approach will work best: epigenomic mapping can complement eQTL analysis for identifying the genes, DNA regulatory sequences, and biological pathways that underlie human traits and diseases. Supporting this approach is recent work by investigators in the IHEC, who have integrated genetic, epigenetic, and transcriptomic profiling across three immune cell types from nearly 200 people [37, 123]. As we know from the ENCODE project, the value of such data can be best realized with the creation of searchable databases of allele-specific epigenetic marks, preferably visualized on sequence tracks such as those in the UCSC Genome Browser [124]. As mQTLs/hap-ASM can be highly tissue-specific, separate tracks for each tissue and cell type are needed. The first steps toward creating these types of web-based resources are being taken (Box 1).

Last, even high-resolution post-GWAS mapping cannot prove causality, so functional assays are needed to evaluate candidate sequences. For a given candidate regulatory sequence identified by post-GWAS approaches, it has now become feasible to use gene-editing approaches, notably CRISPR technology, to create targeted deletions and mutations in a relevant cell type and to assay the effects of such edits on gene expression [125–127]. For example, if the candidate sequence element is a polymorphic TFBS or CTCF-binding site, then the key experiment will be to mutate that site and assay for the predicted changes both in CpG methylation and in the levels of expression of the candidate gene(s) in the haplotype block.

## Relevance of mQTLs and hap-ASM for interpreting EWAS data

EWAS seek to use case-control or cohort designs to detect changes in DNA methylation that result from disease pathology (i.e., from disease progression, rather than genetic susceptibility) and/or environmental factors, such as dietary influences, including over- or under-nutrition, exposures to environmental toxins, and substance abuse, including common situations such as alcohol consumption and cigarette smoking [128, 129]. Issues of experimental design and caveats for EWAS have been discussed in several papers, including some from us [130, 131], but the number of studies completed to date is smaller than for GWAS, and the criteria for calling true-positive “hits” have yet to be standardized. Among the phenotypes that have been investigated are body mass index (BMI) and T2D [132–134], cardiovascular phenotypes [135–137], cigarette smoking [138–140], Alzheimer’s disease (AD) [141–143], autoimmune and inflammatory diseases [144, 145], and neuropsychiatric disorders, including addictive behavior [116, 146]. As pointed out by us and others, because EWAS specifically seek to identify epigenetic changes that are attributable to non-genetic effects, differences in DNA methylation that are produced by inborn genetic factors, that is, mQTLs and hap-ASM, can complicate the interpretation of the results and need to be controlled for [131, 147]. To put it another way, Barfield et al. [148] noted that as the scale of EWAS approaches that of GWAS, population stratification will need to be addressed. This issue boils down to controlling for mQTLs, and Barfield et al. [148] laid out statistical methods to accomplish this task. Similarly, Pan et al. [149] created an R package, called GEM, that can analyze and control for mQTLs and the interaction of genotype and environment (GxE) in EWAS.

To what extent do EWAS hits actually consist of mQTLs? Although most of the EWAS that we have surveyed have not controlled for mQTLs, the recommendations have not been entirely ignored. For example, in their EWAS for BMI, Dick et al. [134] discussed an mQTL-like effect, namely a significant correlation of two SNPs with methylation at a particular CpG dinucleotide without a significant correlation with BMI. In their combined EWAS-mQTL study of cigarette smoking, Qiu et al. [150] identified 43 DM CpGs overlapping with mQTLs. Hedman et al. [135] identified significant *cis*-mQTLs at 64% of the 193 CpGs associated with lipid traits in blood. Likewise, in an EWAS using blood samples, Hannon et al. [116] identified 27 schizophrenia GWAS peaks that had nearby DMRs in schizophrenia versus controls, which co-localized with mQTLs.

To address this question quantitatively, we compiled findings of DM CpGs from multiple EWAS for three important phenotypes: BMI/T2D, AD, and tobacco smoking [132, 133, 138–143, 151–154]. Using the authors’ criteria for statistical significance, we overlapped these EWAS “hits” with mQTL data [49, 54, 62, 108, 110, 112, 113, 150, 155]. We selected the mQTL studies (all included in Table 2) to match the cell types or tissues studied in the EWAS. Among the four EWAS of BMI/T2D that we examined, two utilized PBL samples and two utilized adipose tissue [132, 133, 151, 154]. Of the large number (42,360) of DM CpGs associated with BMI or weight loss in adipose tissue in females, the median differences in methylation were 1.6% per 10 kg.m^2^ BMI and 11.7% per 10 kg.m^2^ BMI before and after weight loss, respectively. Only 2% of these DM CpGs, corresponding to 496 genes, were replicated between the two adipose tissue datasets, which might be partly explained by differences in study design and statistical power. Among these genes, the largest case–control differences were seen for CpGs in *CDR2* and *SEC14L1*, both with differences in methylation of 27% before and after weight loss. In PBL, 400 CpGs were associated with BMI or waist circumference, including 38 CpGs in 27 genes replicated in the two studies. The replicated genes include *LGALS3BP* and *ABCG1*. To assess the proportion of BMI EWAS hits that are mQTLs, we overlapped the BMI DM CpGs in PBL [132] and adipose tissue [133, 151] with blood and adipose tissue mQTL CpGs, respectively. In PBL, we found 48 blood mQTL–EWAS DM overlaps, and 10 SNP-containing CpGs among 400 EWAS DM CpGs, including *HIF3A*. In adipose tissue, we observed a similar percentage of mQTLs among the EWAS DM loci (12.8%, with 4303 EWAS DM CpGs overlapping with mQTLs). Among the 100 top-ranked replicated EWAS DM CpGs (based on difference in methylation), we found that mQTLs could account for 18 of these CpGs, located in ten genes. These genes, including *HIF3A*, *IGFR2*, and *ADSSL1*, will need to be evaluated for their status as bona fide EWAS hits by controlling for the *cis*-acting effects of local haplotypes.

Among the three EWAS of AD that we have reviewed [141–143], none of the 2659 EWAS DM CpGs were reproduced in all datasets. Nevertheless, 0.7% of these DM CpGs, corresponding to 13 genes, were replicated in at least two datasets, including CpGs in *ANK1*, *CDH23*, *SLC44A2*, and *PCNT*. Among these genes, the differences in DNA methylation between cases and controls were small, ranging from 4 to 0.03% [141, 143]. Overall, we observed 85 EWAS DM CpGs that overlapped with brain mQTLs. Thus, at least 5% of the DM CpGs in these AD EWAS might be explained by *cis*-acting genetic effects. These findings are consistent with a recent study that showed that about 5% overlap between schizophrenia DM CpGs and brain mQTLs [156]. However, none of the replicated DM CpGs in the AD EWAS overlapped with mQTLs.

Finally, several EWAS have examined the effects of cigarette smoking on DNA methylation patterns in lung tissue and PBL. mQTL data from lung tissues are sparse, so we focused on the EWAS in PBL. Among five EWAS comparing PBL from current smokers to never smokers [138–140, 152, 153], 18,935 DM CpGs in 6965 genes were identified, with 90% of them showing case–control differences in methylation of less than 1%, but with a small number of loci showing greater DM. A total of 856 CpGs (5%) were replicated as hits in at least two EWAS, and seven genes, including *AHRR*, *GFI1*, *GNA12*, and *LRP5*, were identified as having DM in all five datasets. Once again, the low percentage of replicated EWAS hits might be partly explained by differences in statistical power between studies, with most of the DM being identified only in the large meta-analysis which includes about 16,000 individuals [153]. In contrast to the mild effect sizes seen in AD EWAS, the strongest smoking-associated DM CpGs, in the *AHRR* and *GFI1* genes, showed 24 and 15% differences in methylation, respectively. We found a definite but still relatively modest contribution of *cis*-acting genetic effects among the total EWAS DM CpGs from the five studies, with 3440 CpGs showing a DM–mQTL overlap, as well as 395 SNP-containing CpGs, which together represent 20% of the EWAS DM CpGs. Among the EWAS DM CpGs replicated in at least two studies, there were 12 SNP-containing CpGs and 162 EWAS DM–mQTL overlaps, including CpGs in *AHRR* and *GFI1*, for which a *cis*-effect contribution was shown by Gonseth et al. [157].

On the basis of these findings, we conclude that despite small effect sizes and limited inter-study replication, EWAS have revealed some interesting and reproducible examples of DM, with the majority of published EWAS peaks not being mQTLs. Examples of reproducible and top-ranked DM loci that are not associated with published mQTLs include BMI-associated DM in *HDAC4*, AD-associated DM in *PCNT*, and smoking-associated DM in *F2RL3*. Nonetheless, in our analysis, between 5 and 20% of EWAS DM CpGs overlap with mQTLs. Recently, Chen et al. [37] used a different analytical approach using gene expression as a proxy for disease phenotype and found that *cis*-genetic effects could account for the methylation–expression correlation in more than 50% of the significant genes, suggesting a somewhat higher estimate of genetically influenced loci among EWAS hits.

## Conclusions and future directions

Although GWAS have met part of their initial promise, identifying chromosomal regions that are linked to medically relevant phenotypes, the GWAS design is limited in its ability to pinpoint causal genes and DNA regulatory elements. Genome-wide maps of *cis*-regulated allele-specific phenomena, including eQTLs, mQTLs/hap-ASM, and allele-specific histone modifications and TFBS occupancies, are coming into focus and are helping to nominate candidate genes and DNA sequence variants that can account for GWAS signals. DNA sequence polymorphisms in CTCF and TFBS are emerging as an underlying mechanism for many, but not all, hap-ASM DMRs, and comprehensive efforts to identify these sites are expected to yield insights into transcriptional pathways that affect disease susceptibility.

Nevertheless, a number of challenges still need to be surmounted. As noted above, array-based methods for identifying mQTLs are limited by incomplete and gene-centric coverage, SNPs that can affect probe hybridization, and probes that align to multiple genomic locations [158]. These problems can be solved by using the more direct approaches of targeted and whole genome bis-seq to score ASM. Agilent sequence capture [49], MCC-seq, or WGBS with sample pooling [53] have been employed to achieve sufficient depth, but the newest sequencing platforms are expected to make deep WGBS more practical. As cost will probably remain a factor, it will be useful to determine the optimal sequencing depth for WGBS by performing systematic comparisons with ultra-deep targeted bis-seq [49].

Improvements in epigenomic mapping will also come from the development of more standardized pipelines for data analysis. Basic quality control for methylation BeadChip data, including the filtering of poorly performing probes, normalization and batch adjustment, are well defined [159, 160], but the criteria that define mQTLs are not yet standardized. Approaches to control for the inflation of false positives that results from the high number of correlations being tested are still under investigation [161]. Likewise, in ASM studies, statistical analysis and allele-specific bis-seq alignments are performed using in-house pipelines, in which technical issues, including misalignment of reads mapping to regions with similar bisulfite-converted sequences, achievement of the required depth, bias of the alignments toward the reference allele, and determination of DMRs, have been addressed to varying degrees. More fundamentally, there is already evidence that the knowledge of genotypes at single index SNPs is sometimes insufficient to reveal the haplotype-dependence of ASM—in some instances, the allelic asymmetry can be driven by more than one sequence variant in the local haplotype [49, 57]. This challenge warrants future efforts to determine long-range phased haplotypes. Such efforts can build on conditional analyses [57], SNP phasing approaches [162], and family-based analyses [54]. More directly, sequencing of single DNA molecules to generate bona fide phased genotypes [163] is now being made possible by Illumina (TruSeq® Synthetic Long-read DNA library prep kit).

We believe that it will be important to continue to scrutinize EWAS data for *cis*-acting genetic–epigenetic effects, which need to be filtered out to reveal epigenetic changes that are mediated by the environment or by disease progression, and not by genetics. Conversely, environmental and clinico-demographic factors that are found to associate with DM in EWAS can act as confounders in mQTL/ASM analysis, and will increasingly need to be controlled for as mQTL/ASM studies expand to larger and better-characterized sample groups. Although less directly connected to genetics, changes in cell populations will also need to be more carefully controlled for in EWAS [164]. This caveat is highlighted by findings that DM in *GRP15*, one of the replicated DM loci in smoking EWAS, reflects smoking-induced changes in the composition of T-cell populations [165], and by a meta-analysis showing that some CpGs associated with BMI and eight other cardiometabolic traits are in turn associated with C-reactive protein (CRP) levels, a marker of chronic inflammation [166]. Similarly, the complicating factor of reactive gliosis warrants attention as a possible non-cell-autonomous explanation for the mild DM and low inter-study concordance in AD EWAS.

At the most fundamental level, increasingly thorough mapping of hap-ASM and other allele-specific epigenetic marks in genetically diverse human populations, and in human versus NHP comparisons, will lead to a more complete understanding of the role of allele-specific TFBS occupancies as an underlying mechanism. In this regard, work focusing only on local sequences might fail to reveal a mechanism for all instances of hap-ASM; 3D chromosome architecture will probably need to be taken into account. Future studies can be designed to ask whether some ASM DMRs might be established and propagated based on the presence of rSNPs in TFBSs that are distant from the DMR on a linear scale, but are brought into physical proximity in one or more tissues through chromatin looping (Figs. 2 and 3). This goal of more fully accounting for allele-specific epigenetic patterning in human cells should be achievable by superimposing the locations of ASM DMRs, and allele-specific ATAC-seq and ChIP-seq peaks, onto 3D genome structures elucidated by chromosome conformation capture methods (such as 4C, 5C, and high-throughput chromosome conformation capture [HiC]) or ChIA-PET [122, 167]. Such data will become increasingly useful when centrally compiled, for example, in the 3D Genome Browser [168].

## Box 1 Resources for mapping and analyzing allele-specific epigenetic marks

**Box 1:** Resources for mapping and analyzing allelespecific epigenetic marks

Analytical software	Applications	URL	Reference
Bismark	Bis-seq aligner and methylation caller	http://www.bioinformatics.babraham.ac.uk/projects/bismark/	[169]
BSMAP	Bis-seq aligner	http://lilab.research.bcm.edu/dldcc-web/lilab/yxi/bsmap/bsmap-2.90.tgz	[170]
Bison	Bis-seq aligner and methylation caller	https://github.com/dpryan79/bison	[171]
Bis-SNP	Bis-seq SNP caller	http://people.csail.mit.edu/dnaase/bissnp2011/	[172]
BS-SNPer	Bis-seq SNP caller	https://github.com/hellbelly/BS-Snper	[173]
SNPsplit	Allele-specific alignment sorting	http://www.bioinformatics.babraham.ac.uk/projects/SNPsplit/	[174]
amrfinder	ASM inference from bis-seq	http://smithlabresearch.org/software/amrfinder/	[175]
R package epiG	ASM inference from bis-seq and NOMe-seq data	https://github.com/vincent-dk/epiG	[176]
R package atSNP	Allele-specific transcription factor binding affinity testing	https://github.com/chandlerzuo/atSNP	[177]
Database	Data class	URL	Reference
mQTLdb	mQTL	http://www.mqtldb.org/	[111]
Essex	mQTL	http://epigenetics.essex.ac.uk/mQTL/	[112]
SCAN	mQTL, eQTL	http://www.scandb.org/newinterface/about.html	[178]
SZDB	GWAS, mQTL, eQTL, DM, DE	http://www.szdb.org/index.html	[179]
AlleleDB	ASTF, ASE in LCLs	http://alleledb.gersteinlab.org/	[47]
GRASP	GWAS SNPs, eQTLs, mQTLs, pQTLs, mirQTL	https://grasp.nhlbi.nih.gov/Overview.aspx	[46]
GTEX	eQTLs multiple tissues	https://gtexportal.org/home/	[180]
RegulomeDB	SNP functional annotation (chromatin, TF peaks and binding affinity, DNAse, eQTLs)	http://regulomedb.org/	[20]
SNP2TFBS	SNPs affecting predicted TF binding affinity	http://ccg.vital-it.ch/snp2tfbs/	[181]
Central web sites for human epigenome projects			
NIH Roadmap Epigenomics Project		http://www.roadmapepigenomics.org/	
International Human Epigenome Consortium (IHEC)		http://ihec-epigenomes.org/	

## Abbreviations

AD: Alzheimer’s disease
ASE: Allele-specific expression
ASM: Allele-specific methylation
ASTF: Allele-specific transcription factor
ATAC: Assay for Transposase-Accessible Chromatin
bis-seq: Bisulfite sequencing
BMI: Body mass index
CEPH: Centre d’Etude du Polymorphisme Humain
ChIA-PET: Chromatin interaction analysis by paired-end tag sequencing
ChIP: Chromatin immunoprecipitation
ChIP-seq: ChIP-sequencing
CTCF: CCCTC-binding factor
DM: Differentially methylated
DMR: Differentially methylated region
eQTL: Expression quantitative trait locus
ES: Embryonic stem cell
EWAS: Epigenome-wide association study
FAIRE: Formaldehyde-assisted isolation of regulatory elements
GTEx project: Genotype-Tissue Expression project
GWAS: Genome-wide association study
hap-ASM: Haplotype-dependent allele-specific DNA methylation
IHEC: International Human Epigenome Consortium
LCL: Lymphoblastoid cell line
LD: Linkage disequilibrium
MCC-seq: MethylC-Capture sequencing
mQTL: Methylation quantitative trait locus
MSNP: Methylation-sensitive SNP array
NHP: Non-human primate
PBL: Total peripheral blood
PWM: Position weight matrix
QTL: Quantitative trait locus
rSNP: regulatory SNP
SNP: Single nucleotide or simple nucleotide polymorphism
TAD: Topologically associating domain
TF: Transcription factor
TFBS: Transcription factor binding site
WGBS: Whole genome bisulfite sequencing

## References

[1] Visscher PM, Brown MA, McCarthy MI, Yang J (2012). Five years of GWAS discovery. Am J Hum Genet.

[2] Korf BR (2013). Integration of genomics into medical practice. Discov Med.

[3] Couch FJ, Kuchenbaecker KB, Michailidou K, Mendoza-Fandino GA, Nord S, Lilyquist J (2016). Identification of four novel susceptibility loci for oestrogen receptor negative breast cancer. Nat Commun.

[4] Reeves GK, Travis RC, Green J, Bull D, Tipper S, Baker K (2010). Incidence of breast cancer and its subtypes in relation to individual and multiple low-penetrance genetic susceptibility loci. JAMA.

[5] Muranen TA, Mavaddat N, Khan S, Fagerholm R, Pelttari L, Lee A (2016). Polygenic risk score is associated with increased disease risk in 52 Finnish breast cancer families. Breast Cancer Res Treat.

[6] Sode J, Vogel U, Bank S, Andersen PS, Hetland ML, Locht H (2015). Genetic variations in pattern recognition receptor loci are associated with anti-TNF response in patients with rheumatoid arthritis. PLoS One.

[7] Smith AH, Jensen KP, Li J, Nunez Y, Farrer LA, Hakonarson H (2017). Genome-wide association study of therapeutic opioid dosing identifies a novel locus upstream of OPRM1. Mol Psychiatry.

[8] Zhong H, Yang X, Kaplan LM, Molony C, Schadt EE (2010). Integrating pathway analysis and genetics of gene expression for genome-wide association studies. Am J Hum Genet.

[9] Eichler EE, Flint J, Gibson G, Kong A, Leal SM, Moore JH, Nadeau JH (2010). Missing heritability and strategies for finding the underlying causes of complex disease. Nat Rev Genet.

[10] Flint J (2016). Rare genetic variants and schizophrenia. Nat Neurosci.

[11] Goes FS (2016). Genetics of bipolar disorder: recent update and future directions. Psychiatr Clin North Am.

[12] Visschedijk MC, Alberts R, Mucha S, Deelen P, de Jong DJ, Pierik M (2016). Pooled resequencing of 122 ulcerative colitis genes in a large Dutch cohort suggests population-specific associations of rare variants in MUC2. PLoS One.

[13] Kosmicki JA, Churchhouse CL, Rivas MA, Neale BM (2016). Discovery of rare variants for complex phenotypes. Hum Genet.

[14] Morrison AC, Voorman A, Johnson AD, Liu X, Yu J, Li A (2013). Whole-genome sequence-based analysis of high-density lipoprotein cholesterol. Nat Genet.

[15] Park JH, Gail MH, Weinberg CR, Carroll RJ, Chung CC, Wang Z (2011). Distribution of allele frequencies and effect sizes and their interrelationships for common genetic susceptibility variants. Proc Natl Acad Sci U S A.

[16] Gelernter J, Kranzler HR, Sherva R, Almasy L, Koesterer R, Smith AH (2014). Genome-wide association study of alcohol dependence: significant findings in African- and European-Americans including novel risk loci. Mol Psychiatry.

[17] McPherson R, Tybjaerg-Hansen A (2016). Genetics of coronary artery disease. Circ Res.

[18] Hindorff LA, Sethupathy P, Junkins HA, Ramos EM, Mehta JP, Collins FS, Manolio TA (2009). Potential etiologic and functional implications of genome-wide association loci for human diseases and traits. Proc Natl Acad Sci U S A.

[19] Claussnitzer M, Dankel SN, Kim KH, Quon G, Meuleman W, Haugen C (2015). FTO obesity variant circuitry and adipocyte browning in humans. N Engl J Med.

[20] Boyle AP, Hong EL, Hariharan M, Cheng Y, Schaub MA, Kasowski M (2012). Annotation of functional variation in personal genomes using RegulomeDB. Genome Res.

[21] Schadt EE, Molony C, Chudin E, Hao K, Yang X, Lum PY (2008). Mapping the genetic architecture of gene expression in human liver. PLoS Biol.

[22] Dermitzakis ET, Stranger BE (2006). Genetic variation in human gene expression. Mamm Genome.

[23] Stranger BE, Forrest MS, Clark AG, Minichiello MJ, Deutsch S, Lyle R (2005). Genome-wide associations of gene expression variation in humans. PLoS Genet.

[24] Stranger BE, Forrest MS, Dunning M, Ingle CE, Beazley C, Thorne N (2007). Relative impact of nucleotide and copy number variation on gene expression phenotypes. Science.

[25] Pastinen T, Ge B, Hudson TJ. Influence of human genome polymorphism on gene expression. Hum Mol Genet. 2006;15 Spec No 1:R9–16. 10.1093/hmg/ddl044 16651375

[26] Spielman RS, Bastone LA, Burdick JT, Morley M, Ewens WJ, Cheung VG (2007). Common genetic variants account for differences in gene expression among ethnic groups. Nat Genet.

[27] Schadt EE, Monks SA, Drake TA, Lusis AJ, Che N, Colinayo V (2003). Genetics of gene expression surveyed in maize, mouse and man. Nature.

[28] Cheung VG, Conlin LK, Weber TM, Arcaro M, Jen KY, Morley M, Spielman RS (2003). Natural variation in human gene expression assessed in lymphoblastoid cells. Nat Genet.

[29] Ge B, Pokholok DK, Kwan T, Grundberg E, Morcos L, Verlaan DJ (2009). Global patterns of cis variation in human cells revealed by high-density allelic expression analysis. Nat Genet.

[30] Rotival M, Zeller T, Wild PS, Maouche S, Szymczak S, Schillert A (2011). Integrating genome-wide genetic variations and monocyte expression data reveals trans-regulated gene modules in humans. PLoS Genet.

[31] Zhang X, Gierman HJ, Levy D, Plump A, Dobrin R, Goring HH (2014). Synthesis of 53 tissue and cell line expression QTL datasets reveals master eQTLs. BMC Genomics.

[32] Pastinen T, Hudson TJ (2004). Cis-acting regulatory variation in the human genome. Science.

[33] Heap GA, Yang JH, Downes K, Healy BC, Hunt KA, Bockett N (2010). Genome-wide analysis of allelic expression imbalance in human primary cells by high-throughput transcriptome resequencing. Hum Mol Genet.

[34] Battle A, Mostafavi S, Zhu X, Potash JB, Weissman MM, McCormick C (2014). Characterizing the genetic basis of transcriptome diversity through RNA-sequencing of 922 individuals. Genome Res.

[35] Webster JA, Gibbs JR, Clarke J, Ray M, Zhang W, Holmans P (2009). Genetic control of human brain transcript expression in Alzheimer disease. Am J Hum Genet.

[36] Garnier S, Truong V, Brocheton J, Zeller T, Rovital M, Wild PS (2013). Genome-wide haplotype analysis of cis expression quantitative trait loci in monocytes. PLoS Genet.

[37] Chen L, Ge B, Casale FP, Vasquez L, Kwan T, Garrido-Martin D (2016). Genetic drivers of epigenetic and transcriptional variation in human immune cells. Cell.

[38] Fairfax BP, Humburg P, Makino S, Naranbhai V, Wong D, Lau E (2014). Innate immune activity conditions the effect of regulatory variants upon monocyte gene expression. Science.

[39] Peters JE, Lyons PA, Lee JC, Richard AC, Fortune MD, Newcombe PJ (2016). Insight into genotype-phenotype associations through eQTL mapping in multiple cell types in health and immune-mediated disease. PLoS Genet.

[40] Nica AC, Montgomery SB, Dimas AS, Stranger BE, Beazley C, Barroso I, Dermitzakis ET (2010). Candidate causal regulatory effects by integration of expression QTLs with complex trait genetic associations. PLoS Genet.

[41] Zhong H, Beaulaurier J, Lum PY, Molony C, Yang X, Macneil DJ (2010). Liver and adipose expression associated SNPs are enriched for association to type 2 diabetes. PLoS Genet.

[42] Chun S, Casparino A, Patsopoulos NA, Croteau-Chonka DC, Raby BA, De Jager PL (2017). Limited statistical evidence for shared genetic effects of eQTLs and autoimmune-disease-associated loci in three major immune-cell types. Nat Genet.

[43] Fortune MD, Guo H, Burren O, Schofield E, Walker NM, Ban M (2015). Statistical colocalization of genetic risk variants for related autoimmune diseases in the context of common controls. Nat Genet.

[44] Guo H, Fortune MD, Burren OS, Schofield E, Todd JA, Wallace C (2015). Integration of disease association and eQTL data using a Bayesian colocalisation approach highlights six candidate causal genes in immune-mediated diseases. Hum Mol Genet.

[45] Leslie R, O'Donnell CJ, Johnson AD (2014). GRASP: analysis of genotype-phenotype results from 1390 genome-wide association studies and corresponding open access database. Bioinformatics.

[46] Eicher JD, Landowski C, Stackhouse B, Sloan A, Chen W, Jensen N (2015). GRASP v2.0: an update on the genome-wide repository of associations between SNPs and phenotypes. Nucleic Acids Res.

[47] Chen J, Rozowsky J, Galeev TR, Harmanci A, Kitchen R, Bedford J (2016). A uniform survey of allele-specific binding and expression over 1000-Genomes-Project individuals. Nat Commun.

[48] Kerkel K, Spadola A, Yuan E, Kosek J, Jiang L, Hod E (2008). Genomic surveys by methylation-sensitive SNP analysis identify sequence-dependent allele-specific DNA methylation. Nat Genet.

[49] Do C, Lang CF, Lin J, Darbary H, Krupska I, Gaba A (2016). Mechanisms and disease associations of haplotype-dependent allele-specific DNA methylation. Am J Hum Genet.

[50] Glaser RL, Ramsay JP, Morison IM (2006). The imprinted gene and parent-of-origin effect database now includes parental origin of de novo mutations. Nucleic Acids Res.

[51] Paliwal A, Temkin AM, Kerkel K, Yale A, Yotova I, Drost N (2013). Comparative anatomy of chromosomal domains with imprinted and non-imprinted allele-specific DNA methylation. PLoS Genet.

[52] Das R, Lee YK, Strogantsev R, Jin S, Lim YC, Ng PY (2013). DNMT1 and AIM1 imprinting in human placenta revealed through a genome-wide screen for allele-specific DNA methylation. BMC Genomics.

[53] Cheung WA, Shao X, Morin A, Siroux V, Kwan T, Ge B (2017). Functional variation in allelic methylomes underscores a strong genetic contribution and reveals novel epigenetic alterations in the human epigenome. Genome Biol.

[54] Day K, Waite LL, Alonso A, Irvin MR, Zhi D, Thibeault KS (2016). Heritable DNA methylation in CD4+ cells among complex families displays genetic and non-genetic effects. PLoS One.

[55] Olsson AH, Volkov P, Bacos K, Dayeh T, Hall E, Nilsson EA (2014). Genome-wide associations between genetic and epigenetic variation influence mRNA expression and insulin secretion in human pancreatic islets. PLoS Genet.

[56] Quilez J, Guilmatre A, Garg P, Highnam G, Gymrek M, Erlich Y (2016). Polymorphic tandem repeats within gene promoters act as modifiers of gene expression and DNA methylation in humans. Nucleic Acids Res.

[57] Richardson TG, Shihab HA, Hemani G, Zheng J, Hannon E, Mill J (2016). Collapsed methylation quantitative trait loci analysis for low frequency and rare variants. Hum Mol Genet.

[58] McClay JL, Shabalin AA, Dozmorov MG, Adkins DE, Kumar G, Nerella S (2015). High density methylation QTL analysis in human blood via next-generation sequencing of the methylated genomic DNA fraction. Genome Biol.

[59] Shoemaker R, Deng J, Wang W, Zhang K (2010). Allele-specific methylation is prevalent and is contributed by CpG-SNPs in the human genome. Genome Res.

[60] Li Y, Zhu J, Tian G, Li N, Li Q, Ye M (2010). The DNA methylome of human peripheral blood mononuclear cells. PLoS Biol.

[61] Gertz J, Varley KE, Reddy TE, Bowling KM, Pauli F, Parker SL (2011). Analysis of DNA methylation in a three-generation family reveals widespread genetic influence on epigenetic regulation. PLoS Genet.

[62] Zhang D, Cheng L, Badner JA, Chen C, Chen Q, Luo W (2010). Genetic control of individual differences in gene-specific methylation in human brain. Am J Hum Genet.

[63] Hutchinson JN, Raj T, Fagerness J, Stahl E, Viloria FT, Gimelbrant A (2014). Allele-specific methylation occurs at genetic variants associated with complex disease. PLoS One.

[64] Williams A, Flavell RA (2008). The role of CTCF in regulating nuclear organization. J Exp Med.

[65] Ong CT, Corces VG (2014). CTCF: an architectural protein bridging genome topology and function. Nat Rev Genet.

[66] Bell AC, Felsenfeld G (2000). Methylation of a CTCF-dependent boundary controls imprinted expression of the *Igf2* gene. Nature.

[67] Hark AT, Schoenherr CJ, Katz DJ, Ingram RS, Levorse JM, Tilghman SM (2000). CTCF mediates methylation-sensitive enhancer-blocking activity at the H19/Igf2 locus. Nature.

[68] Takai D, Gonzales FA, Tsai YC, Thayer MJ, Jones PA (2001). Large scale mapping of methylcytosines in CTCF-binding sites in the human H19 promoter and aberrant hypomethylation in human bladder cancer. Hum Mol Genet.

[69] Kemp CJ, Moore JM, Moser R, Bernard B, Teater M, Smith LE (2014). CTCF haploinsufficiency destabilizes DNA methylation and predisposes to cancer. Cell Rep.

[70] Cooper CD, Newman JA, Aitkenhead H, Allerston CK, Gileadi O (2015). Structures of the Ets protein DNA-binding domains of transcription factors Etv1, Etv4, Etv5, and Fev: determinants of DNA binding and redox regulation by disulfide bond formation. J Biol Chem.

[71] Stephens DC, Poon GM (2016). Differential sensitivity to methylated DNA by ETS-family transcription factors is intrinsically encoded in their DNA-binding domains. Nucleic Acids Res.

[72] Reynard LN, Bui C, Syddall CM, Loughlin J (2014). CpG methylation regulates allelic expression of GDF5 by modulating binding of SP1 and SP3 repressor proteins to the osteoarthritis susceptibility SNP rs143383. Hum Genet.

[73] Boumber YA, Kondo Y, Chen X, Shen L, Guo Y, Tellez C (2008). An Sp1/Sp3 binding polymorphism confers methylation protection. PLoS Genet.

[74] Stadler MB, Murr R, Burger L, Ivanek R, Lienert F, Scholer A (2011). DNA-binding factors shape the mouse methylome at distal regulatory regions. Nature.

[75] Xu T, Li B, Zhao M, Szulwach KE, Street RC, Lin L (2015). Base-resolution methylation patterns accurately predict transcription factor bindings in vivo. Nucleic Acids Res.

[76] Feldmann A, Ivanek R, Murr R, Gaidatzis D, Burger L, Schubeler D (2013). Transcription factor occupancy can mediate active turnover of DNA methylation at regulatory regions. PLoS Genet.

[77] Anvar Z, Cammisa M, Riso V, Baglivo I, Kukreja H, Sparago A (2016). ZFP57 recognizes multiple and closely spaced sequence motif variants to maintain repressive epigenetic marks in mouse embryonic stem cells. Nucleic Acids Res.

[78] Hu S, Wan J, Su Y, Song Q, Zeng Y, Nguyen HN (2013). DNA methylation presents distinct binding sites for human transcription factors. Elife.

[79] Yin Y, Morgunova E, Jolma A, Kaasinen E, Sahu B, Khund-Sayeed S, et al. Impact of cytosine methylation on DNA binding specificities of human transcription factors. Science. 2017;356. doi: 10.1126/science.aaj2239. [Epub ahead of print] 10.1126/science.aaj2239 PMC8009048 28473536

[80] Jia D, Jurkowska RZ, Zhang X, Jeltsch A, Cheng X (2007). Structure of Dnmt3a bound to Dnmt3L suggests a model for de novo DNA methylation. Nature.

[81] Glass JL, Fazzari MJ, Ferguson-Smith AC, Greally JM (2009). CG dinucleotide periodicities recognized by the Dnmt3a-Dnmt3L complex are distinctive at retroelements and imprinted domains. Mamm Genome.

[82] Du Q, Luu PL, Stirzaker C, Clark SJ (2015). Methyl-CpG-binding domain proteins: readers of the epigenome. Epigenomics.

[83] Tycko B (2010). Allele-specific DNA, methylation: beyond imprinting. Hum Mol Genet.

[84] Knight JC, Keating BJ, Rockett KA, Kwiatkowski DP (2003). In vivo characterization of regulatory polymorphisms by allele-specific quantification of RNA polymerase loading. Nat Genet.

[85] Kadota M, Yang HH, Hu N, Wang C, Hu Y, Taylor PR (2007). Allele-specific chromatin immunoprecipitation studies show genetic influence on chromatin state in human genome. PLoS Genet.

[86] Maynard ND, Chen J, Stuart RK, Fan JB, Ren B (2008). Genome-wide mapping of allele-specific protein-DNA interactions in human cells. Nat Methods.

[87] Kasowski M, Grubert F, Heffelfinger C, Hariharan M, Asabere A, Waszak SM (2010). Variation in transcription factor binding among humans. Science.

[88] McDaniell R, Lee BK, Song L, Liu Z, Boyle AP, Erdos MR (2010). Heritable individual-specific and allele-specific chromatin signatures in humans. Science.

[89] Kim K, Ban HJ, Seo J, Lee K, Yavartanoo M, Kim SC (2014). Genetic factors underlying discordance in chromatin accessibility between monozygotic twins. Genome Biol.

[90] Reddy TE, Gertz J, Pauli F, Kucera KS, Varley KE, Newberry KM (2012). Effects of sequence variation on differential allelic transcription factor occupancy and gene expression. Genome Res.

[91] Lu X, Zoller EE, Weirauch MT, Wu Z, Namjou B, Williams AH (2015). Lupus risk variant increases pSTAT1 binding and decreases ETS1 expression. Am J Hum Genet.

[92] Cavalli M, Pan G, Nord H, Wallen Arzt E, Wallerman O, Wadelius C (2016). Allele-specific transcription factor binding in liver and cervix cells unveils many likely drivers of GWAS signals. Genomics.

[93] Pai AA, Pritchard JK, Gilad Y (2015). The genetic and mechanistic basis for variation in gene regulation. PLoS Genet.

[94] Butter F, Davison L, Viturawong T, Scheibe M, Vermeulen M, Todd JA, Mann M (2012). Proteome-wide analysis of disease-associated SNPs that show allele-specific transcription factor binding. PLoS Genet.

[95] Banovich NE, Lan X, McVicker G, van de Geijn B, Degner JF, Blischak JD (2014). Methylation QTLs are associated with coordinated changes in transcription factor binding, histone modifications, and gene expression levels. PLoS Genet.

[96] Schilling E, El Chartouni C, Rehli M (2009). Allele-specific DNA methylation in mouse strains is mainly determined by cis-acting sequences. Genome Res.

[97] Wetterbom A, Sevov M, Cavelier L, Bergstrom TF (2006). Comparative genomic analysis of human and chimpanzee indicates a key role for indels in primate evolution. J Mol Evol.

[98] Wooding S, Jorde LB (2006). Duplication and divergence in humans and chimpanzees. Bioessays.

[99] Cheng Z, Ventura M, She X, Khaitovich P, Graves T, Osoegawa K (2005). A genome-wide comparison of recent chimpanzee and human segmental duplications. Nature.

[100] Hernando-Herraez I, Prado-Martinez J, Garg P, Fernandez-Callejo M, Heyn H, Hvilsom C (2013). Dynamics of DNA methylation in recent human and great ape evolution. PLoS Genet.

[101] Zeng J, Konopka G, Hunt BG, Preuss TM, Geschwind D, Yi SV (2012). Divergent whole-genome methylation maps of human and chimpanzee brains reveal epigenetic basis of human regulatory evolution. Am J Hum Genet.

[102] Farh KK, Marson A, Zhu J, Kleinewietfeld M, Housley WJ, Beik S (2015). Genetic and epigenetic fine mapping of causal autoimmune disease variants. Nature.

[103] Lupianez DG, Spielmann M, Mundlos S (2016). Breaking TADs: how alterations of chromatin domains result in disease. Trends Genet.

[104] Tycko B (2010). Mapping allele-specific DNA methylation: a new tool for maximizing information from GWAS. Am J Hum Genet.

[105] Meaburn EL, Schalkwyk LC, Mill J (2010). Allele-specific methylation in the human genome: implications for genetic studies of complex disease. Epigenetics.

[106] Zhang H, Wang F, Kranzler HR, Yang C, Xu H, Wang Z (2014). Identification of methylation quantitative trait loci (mQTLs) influencing promoter DNA methylation of alcohol dependence risk genes. Hum Genet.

[107] Kato N, Loh M, Takeuchi F, Verweij N, Wang X, Zhang W (2015). Trans-ancestry genome-wide association study identifies 12 genetic loci influencing blood pressure and implicates a role for DNA methylation. Nat Genet.

[108] Gibbs JR, van der Brug MP, Hernandez DG, Traynor BJ, Nalls MA, Lai SL (2010). Abundant quantitative trait loci exist for DNA methylation and gene expression in human brain. PLoS Genet.

[109] Gamazon ER, Badner JA, Cheng L, Zhang C, Zhang D, Cox NJ (2013). Enrichment of cis-regulatory gene expression SNPs and methylation quantitative trait loci among bipolar disorder susceptibility variants. Mol Psychiatry.

[110] Grundberg E, Meduri E, Sandling JK, Hedman AK, Keildson S, Buil A (2013). Global analysis of DNA methylation variation in adipose tissue from twins reveals links to disease-associated variants in distal regulatory elements. Am J Hum Genet.

[111] Gaunt TR, Shihab HA, Hemani G, Min JL, Woodward G, Lyttleton O (2016). Systematic identification of genetic influences on methylation across the human life course. Genome Biol.

[112] Hannon E, Spiers H, Viana J, Pidsley R, Burrage J, Murphy TM (2016). Methylation QTLs in the developing brain and their enrichment in schizophrenia risk loci. Nat Neurosci.

[113] Volkov P, Olsson AH, Gillberg L, Jorgensen SW, Brons C, Eriksson KF (2016). A genome-wide mQTL analysis in human adipose tissue identifies genetic variants associated with DNA methylation, gene expression and metabolic traits. PLoS One.

[114] Wagner JR, Busche S, Ge B, Kwan T, Pastinen T, Blanchette M (2014). The relationship between DNA methylation, genetic and expression inter-individual variation in untransformed human fibroblasts. Genome Biol.

[115] Gutierrez-Arcelus M, Ongen H, Lappalainen T, Montgomery SB, Buil A, Yurovsky A (2015). Tissue-specific effects of genetic and epigenetic variation on gene regulation and splicing. PLoS Genet.

[116] Hannon E, Dempster E, Viana J, Burrage J, Smith AR, Macdonald R (2016). An integrated genetic-epigenetic analysis of schizophrenia: evidence for co-localization of genetic associations and differential DNA methylation. Genome Biol.

[117] Kumasaka N, Knights AJ, Gaffney DJ (2016). Fine-mapping cellular QTLs with RASQUAL and ATAC-seq. Nat Genet.

[118] Scott LJ, Erdos MR, Huyghe JR, Welch RP, Beck AT, Wolford BN (2016). The genetic regulatory signature of type 2 diabetes in human skeletal muscle. Nat Commun.

[119] Abdul-Ghani MA, DeFronzo RA (2010). Pathogenesis of insulin resistance in skeletal muscle. J Biomed Biotechnol.

[120] de Santiago I, Liu W, Yuan K, O'Reilly M, Chilamakuri CS, Ponder BA (2017). BaalChIP: Bayesian analysis of allele-specific transcription factor binding in cancer genomes. Genome Biol.

[121] Smith AJ, Howard P, Shah S, Eriksson P, Stender S, Giambartolomei C (2012). Use of allele-specific FAIRE to determine functional regulatory polymorphism using large-scale genotyping arrays. PLoS Genet.

[122] Tang Z, Luo OJ, Li X, Zheng M, Zhu JJ, Szalaj P (2015). CTCF-mediated human 3D genome architecture reveals chromatin topology for transcription. Cell.

[123] Astle WJ, Elding H, Jiang T, Allen D, Ruklisa D, Mann AL (2016). The allelic landscape of human blood cell trait variation and links to common complex disease. Cell.

[124] Kent WJ, Sugnet CW, Furey TS, Roskin KM, Pringle TH, Zahler AM, Haussler D (2002). The human genome browser at UCSC. Genome Res.

[125] Liu XS, Wu H, Ji X, Stelzer Y, Wu X, Czauderna S (2016). Editing DNA methylation in the mammalian genome. Cell.

[126] Ji X, Dadon DB, Powell BE, Fan ZP, Borges-Rivera D, Shachar S (2016). 3D chromosome regulatory landscape of human pluripotent cells. Cell Stem Cell.

[127] Hnisz D, Weintraub AS, Day DS, Valton AL, Bak RO, Li CH (2016). Activation of proto-oncogenes by disruption of chromosome neighborhoods. Science.

[128] Philibert R, Erwin C (2015). A review of epigenetic markers of tobacco and alcohol consumption. Behav Sci Law.

[129] Liu C, Marioni RE, Hedman AK, Pfeiffer L, Tsai PC, Reynolds LM (2016). A DNA methylation biomarker of alcohol consumption. Mol Psychiatry.

[130] Hatchwell E, Greally JM (2007). The potential role of epigenomic dysregulation in complex human disease. Trends Genet.

[131] Michels KB, Binder AM, Dedeurwaerder S, Epstein CB, Greally JM, Gut I (2013). Recommendations for the design and analysis of epigenome-wide association studies. Nat Methods.

[132] Demerath EW, Guan W, Grove ML, Aslibekyan S, Mendelson M, Zhou YH (2015). Epigenome-wide association study (EWAS) of BMI, BMI change and waist circumference in African American adults identifies multiple replicated loci. Hum Mol Genet.

[133] Ronn T, Volkov P, Gillberg L, Kokosar M, Perfilyev A, Jacobsen AL (2015). Impact of age, BMI and HbA1c levels on the genome-wide DNA methylation and mRNA expression patterns in human adipose tissue and identification of epigenetic biomarkers in blood. Hum Mol Genet.

[134] Dick KJ, Nelson CP, Tsaprouni L, Sandling JK, Aïssi D, Wahl S (2014). DNA methylation and body-mass index: a genome-wide analysis. Lancet.

[135] Hedman AK, Mendelson MM, Marioni RE, Gustafsson S, Joehanes R, Irvin MR, et al. Epigenetic patterns in blood associated with lipid traits predict incident coronary heart disease events and are enriched for results from genome-wide association studies. Circ Cardiovasc Genet. 2017;10. doi: 10.1161/CIRCGENETICS.116.001487. [Epub ahead of print] 10.1161/CIRCGENETICS.116.001487 PMC5331877 28213390

[136] Li J, Zhu X, Yu K, Jiang H, Zhang Y, Deng S (2017). Genome-wide analysis of DNA methylation and acute coronary syndrome. Circ Res.

[137] Zhang J, Liu Z, Umukoro PE, Cavallari JM, Fang SC, Weisskopf MG (2017). An epigenome-wide association analysis of cardiac autonomic responses among a population of welders. Epigenetics.

[138] Zeilinger S, Kuhnel B, Klopp N, Baurecht H, Kleinschmidt A, Gieger C (2013). Tobacco smoking leads to extensive genome-wide changes in DNA methylation. PLoS One.

[139] Shenker NS, Polidoro S, van Veldhoven K, Sacerdote C, Ricceri F, Birrell MA (2013). Epigenome-wide association study in the European Prospective Investigation into Cancer and Nutrition (EPIC-Turin) identifies novel genetic loci associated with smoking. Hum Mol Genet.

[140] Guida F, Sandanger TM, Castagne R, Campanella G, Polidoro S, Palli D (2015). Dynamics of smoking-induced genome-wide methylation changes with time since smoking cessation. Hum Mol Genet.

[141] Lunnon K, Smith R, Hannon E, De Jager PL, Srivastava G, Volta M (2014). Methylomic profiling implicates cortical deregulation of ANK1 in Alzheimer's disease. Nat Neurosci.

[142] De Jager PL, Srivastava G, Lunnon K, Burgess J, Schalkwyk LC, Yu L (2014). Alzheimer's disease: early alterations in brain DNA methylation at ANK1, BIN1, RHBDF2 and other loci. Nat Neurosci.

[143] Watson CT, Roussos P, Garg P, Ho DJ, Azam N, Katsel PL (2016). Genome-wide DNA methylation profiling in the superior temporal gyrus reveals epigenetic signatures associated with Alzheimer's disease. Genome Med.

[144] Li Yim AY, Duijvis NW, Zhao J, de Jonge WJ, D'Haens GR, Mannens MM (2016). Peripheral blood methylation profiling of female Crohn's disease patients. Clin Epigenetics.

[145] Zimmermann MT, Oberg AL, Grill DE, Ovsyannikova IG, Haralambieva IH, Kennedy RB, Poland GA (2016). System-wide associations between DNA-methylation, gene expression, and humoral immune response to influenza vaccination. PLoS One.

[146] Zhang R, Miao Q, Wang C, Zhao R, Li W, Haile CN (2013). Genome-wide DNA methylation analysis in alcohol dependence. Addict Biol.

[147] Rakyan VK, Down TA, Balding DJ, Beck S (2011). Epigenome-wide association studies for common human diseases. Nat Rev Genet.

[148] Barfield RT, Almli LM, Kilaru V, Smith AK, Mercer KB, Duncan R (2014). Accounting for population stratification in DNA methylation studies. Genet Epidemiol.

[149] Pan H, Holbrook JD, Karnani N, Kwoh CK (2016). Gene, Environment and Methylation (GEM): a tool suite to efficiently navigate large scale epigenome wide association studies and integrate genotype and interaction between genotype and environment. BMC Bioinformatics.

[150] Qiu W, Wan E, Morrow J, Cho MH, Crapo JD, Silverman EK, DeMeo DL (2015). The impact of genetic variation and cigarette smoke on DNA methylation in current and former smokers from the COPDGene study. Epigenetics.

[151] Benton MC, Johnstone A, Eccles D, Harmon B, Hayes MT, Lea RA (2015). An analysis of DNA methylation in human adipose tissue reveals differential modification of obesity genes before and after gastric bypass and weight loss. Genome Biol.

[152] Ambatipudi S, Cuenin C, Hernandez-Vargas H, Ghantous A, Le Calvez-Kelm F, Kaaks R (2016). Tobacco smoking-associated genome-wide DNA methylation changes in the EPIC study. Epigenomics.

[153] Joehanes R, Just AC, Marioni RE, Pilling LC, Reynolds LM, Mandaviya PR (2016). Epigenetic signatures of cigarette smoking. Circ Cardiovasc Genet.

[154] Wahl S, Drong A, Lehne B, Loh M, Scott WR, Kunze S (2017). Epigenome-wide association study of body mass index, and the adverse outcomes of adiposity. Nature.

[155] Smith AK, Kilaru V, Kocak M, Almli LM, Mercer KB, Ressler KJ (2014). Methylation quantitative trait loci (meQTLs) are consistently detected across ancestry, developmental stage, and tissue type. BMC Genomics.

[156] Jaffe AE, Gao Y, Deep-Soboslay A, Tao R, Hyde TM, Weinberger DR, Kleinman JE (2016). Mapping DNA methylation across development, genotype and schizophrenia in the human frontal cortex. Nat Neurosci.

[157] Gonseth S, de Smith AJ, Roy R, Zhou M, Lee ST, Shao X (2016). Genetic contribution to variation in DNA methylation at maternal smoking-sensitive loci in exposed neonates. Epigenetics.

[158] Price ME, Cotton AM, Lam LL, Farre P, Emberly E, Brown CJ (2013). Additional annotation enhances potential for biologically-relevant analysis of the Illumina Infinium HumanMethylation450 BeadChip array. Epigenet Chromatin.

[159] Fortin JP, Triche TJ, Hansen KD (2017). Preprocessing, normalization and integration of the Illumina HumanMethylationEPIC array with minfi. Bioinformatics.

[160] Morris TJ, Beck S (2015). Analysis pipelines and packages for Infinium HumanMethylation450 BeadChip (450 k) data. Methods.

[161] Luijk R, Goeman JJ, Slagboom EP, Heijmans BT, van Zwet EW (2015). An alternative approach to multiple testing for methylation QTL mapping reduces the proportion of falsely identified CpGs. Bioinformatics.

[162] Browning SR, Browning BL (2011). Haplotype phasing: existing methods and new developments. Nat Rev Genet.

[163] Kuleshov V, Xie D, Chen R, Pushkarev D, Ma Z, Blauwkamp T (2014). Whole-genome haplotyping using long reads and statistical methods. Nat Biotechnol.

[164] Jaffe AE, Irizarry RA (2014). Accounting for cellular heterogeneity is critical in epigenome-wide association studies. Genome Biol.

[165] Bauer M, Linsel G, Fink B, Offenberg K, Hahn AM, Sack U (2015). A varying T cell subtype explains apparent tobacco smoking induced single CpG hypomethylation in whole blood. Clin Epigenet.

[166] Ligthart S, Marzi C, Aslibekyan S, Mendelson MM, Conneely KN, Tanaka T (2016). DNA methylation signatures of chronic low-grade inflammation are associated with complex diseases. Genome Biol.

[167] Schmitt AD, Hu M, Ren B (2016). Genome-wide mapping and analysis of chromosome architecture. Nat Rev Mol Cell Biol.

[168] Wang Y, Zhang B, Zhang L, An L, Xu J, Li D, et al. The 3D Genome Browser: a web-based browser for visualizing 3D genome organization and long-range chromatin interactions. Biorxiv 2017. doi: https://doi.org/10.1101/112268. [Epub ahead of print] 10.1186/s13059-018-1519-9 PMC6172833 30286773

[169] Krueger F, Andrews SR (2011). Bismark: a flexible aligner and methylation caller for Bisulfite-Seq applications. Bioinformatics.

[170] Xi Y, Li W (2009). BSMAP: whole genome bisulfite sequence MAPping program. BMC Bioinformatics.

[171] Ryan DP, Ehninger D (2014). Bison: bisulfite alignment on nodes of a cluster. BMC Bioinformatics.

[172] Liu Y, Siegmund KD, Laird PW, Berman BP (2012). Bis-SNP: combined DNA methylation and SNP calling for Bisulfite-seq data. Genome Biol.

[173] Gao S, Zou D, Mao L, Liu H, Song P, Chen Y (2015). BS-SNPer: SNP calling in bisulfite-seq data. Bioinformatics.

[174] Krueger F, Andrews SR (2016). SNPsplit: allele-specific splitting of alignments between genomes with known SNP genotypes. F1000Res.

[175] Fang F, Hodges E, Molaro A, Dean M, Hannon GJ, Smith AD (2012). Genomic landscape of human allele-specific DNA methylation. Proc Natl Acad Sci U S A.

[176] Vincent M, Mundbjerg K, Skou Pedersen J, Liang G, Jones PA, Orntoft TF (2017). epiG: statistical inference and profiling of DNA methylation from whole-genome bisulfite sequencing data. Genome Biol.

[177] Zuo C, Shin S, Keles S (2015). atSNP: transcription factor binding affinity testing for regulatory SNP detection. Bioinformatics.

[178] Zhang W, Gamazon ER, Zhang X, Konkashbaev A, Liu C, Szilagyi KL (2015). SCAN database: facilitating integrative analyses of cytosine modification and expression QTL. Database (Oxford).

[179] Wu Y, Yao YG, Luo XJ (2017). SZDB: a database for schizophrenia genetic research. Schizophr Bull.

[180] GTEx Consortium (2013). The Genotype-Tissue Expression (GTEx) project. Nat Genet.

[181] Kumar S, Ambrosini G, Bucher P (2017). SNP2TFBS—a database of regulatory SNPs affecting predicted transcription factor binding site affinity. Nucleic Acids Res.

[182] Zhang Y, Rohde C, Reinhardt R, Voelcker-Rehage C, Jeltsch A (2009). Non-imprinted allele-specific DNA methylation on human autosomes. Genome Biol.

[183] Hellman A, Chess A (2010). Extensive sequence-influenced DNA methylation polymorphism in the human genome. Epigenet Chromatin.

[184] Schalkwyk LC, Meaburn EL, Smith R, Dempster EL, Jeffries AR, Davies MN (2010). Allelic skewing of DNA methylation is widespread across the genome. Am J Hum Genet.

[185] Plongthongkum N, van Eijk KR, de Jong S, Wang T, Sul JH, Boks MP (2014). Characterization of genome-methylome interactions in 22 nuclear pedigrees. PLoS One.

[186] Auton A, Brooks LD, Durbin RM, Garrison EP, Kang HM, 1000 Genomes Project Consortium (2015). A global reference for human genetic variation. Nature.

[187] Purcell S, Neale B, Todd-Brown K, Thomas L, Ferreira MA, Bender D (2007). PLINK: a tool set for whole-genome association and population-based linkage analyses. Am J Hum Genet.

[188] Gabriel SB, Schaffner SF, Nguyen H, Moore JM, Roy J, Blumenstiel B (2002). The structure of haplotype blocks in the human genome. Science.

[189] Petrascheck M, Escher D, Mahmoudi T, Verrijzer CP, Schaffner W, Barberis A (2005). DNA looping induced by a transcriptional enhancer in vivo. Nucleic Acids Res.

